# Residency matches of MD-PhD versus MD-only students: compatibility with continued research momentum

**DOI:** 10.1172/jci.insight.202606

**Published:** 2026-06-22

**Authors:** Shannon Baker, S. Mehdi Nouraie, Richard A. Steinman

**Affiliations:** 1 University of Pittsburgh, Pittsburgh, Pennsylvania, USA.; 2 Division of Pulmonary, Allergy, Critical Care and Sleep Medicine, Department of Medicine,; 3 Department of Medicine, and; 4 Department of Pharmacology & Chemical Biology, University of Pittsburgh School of Medicine, Pittsburgh, Pennsylvania, USA.

## Abstract

Programs at the medical school level to train students as physician-scientists include NIH-supported MD-PhD (Medical Scientist Training Program, MSTP), non–NIH-supported MD-PhD (MDPhD), and limited research pathways for MD-only students. Continued exposure to a research-rich culture in residency could sustain commitment to a physician-scientist career. We compared residency matches of 10,668 MSTP, MDPhD, or MD-only students from 2021–2023 match cycles, using the NIH funding level of the matched residency program’s department in the match year as the central measure. We also measured how medical school NIH funding levels influenced student matches to top-funded residency programs. Public individual-level match data were available from 13 top-NIH-funded medical schools (top quintile, highest 20% funding) and 8 mid-NIH-funded medical schools (mid quintile, 40%–60% funding). Top-quintile medical schools housed only MSTP programs, whereas mid-quintile schools had both MSTP and MDPhD programs. Across all medical schools, MSTP students matched at a higher rate than MD-only students to residencies in top-NIH-funded departments in their discipline. Within the mid-quintile schools, MSTP and MDPhD student matches did not differ significantly. Notably, students from the top-quintile medical schools (both MD-only and MSTP) matched to higher-NIH-funded residency departments than comparably trained students from the mid-quintile schools.

## Graphical abstract

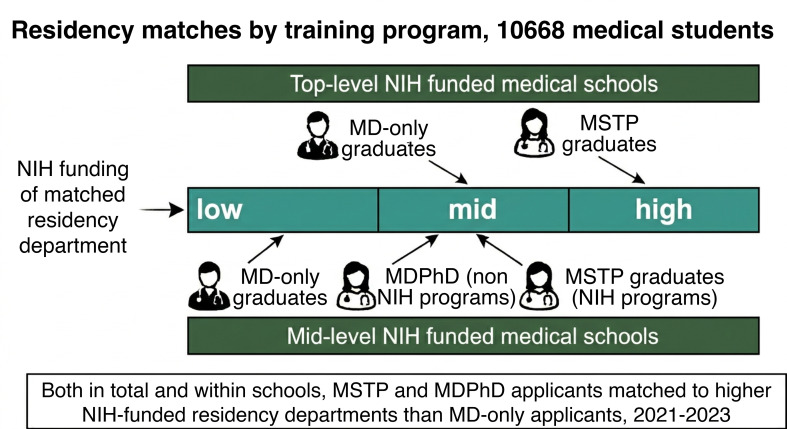

## Introduction

Despite rapid advances in research, there remains a persistent shortage of professionals who can truly integrate clinical experience with scientific expertise. Pregraduate research-intensive MD-PhD training in medical school through NIH-supported Medical Scientist Training Programs (MSTPs), as well as non–NIH-supported MD-PhD programs, comprises a critical source of well-trained physician-scientists. Nonetheless, persistence in the physician-scientist track after graduation is suboptimal, with estimates of 15%–30% attrition among MSTP graduates (1–4) and greater attrition among women physician-scientists (5).

Career transitions are periods in which commitments to a physician-scientist path can waver. Residency training is felt to be one leaky (albeit not quantified) point in the pipeline. Concerns about attrition arising from leaving the research environment for full-time clinical training (3) prompted new intraresidency NIH R38 funding mechanisms (6). Similarly, structured postgraduate Physician Scientist Training Programs (PSTPs) are predicted to facilitate sustained research commitment (7). The aggregate enrollment in these programs is limited and controlled data on the impact of these programs on persistence in the physician-scientist path is not yet available. More broadly, being immersed in a research-positive culture at the residency training site could support trainees’ research passion throughout the residency transition. That culture depends on the postgraduate environment having sufficient research resources, projects, investigator role models, and mentors.

An important facilitator of MD-PhD students continuing as physician-scientists could be their matching to residencies in departments that receive a high level of NIH research funding. Disproportionate matching of MD-PhD students to residencies linked to high NIH funding could indicate that the students are actively seeking a research-rich postgraduate environment. It could also indicate a particular interest on the part of those residency programs in trainees committed to the physician-scientist path.

As a first step in exploring the residency match as a factor in long-term research productivity, we measured how MSTP and non-MSTP MD-PhD students matched to specialties at top-NIH-supported residency sites compared with MD-only students. Hereafter, for ease of reference, we refer to MD-PhD students and programs from NIH-supported programs as “MSTPs” and those from non–NIH-supported programs as “MDPhDs.” Our goals were to (a) determine whether MSTP, MDPhD, or MD students differed in their rates of matching to top-NIH-funded residencies and (b) determine whether the level of NIH support of the medical school that students attended correlated with their match to top-NIH-funded residencies.

The importance of our study is not the genesis of the match decision, but simply whether the trajectory of research-track medical school trainees continues into a research-rich postgraduate environment.

Our study enabled a comparison of match outcomes between schools offering MDPhD training that had distinct levels of NIH support. The Blue Ridge Institute ranks US medical schools annually based on total NIH research funding. In 2023, 144 medical schools were ranked. To facilitate comparative analysis, these institutions were stratified into quintiles, each representing 20% of the ranked medical schools. The level of NIH support for the medical schools was determined based on quintiles of NIH support listed for medical schools by the Blue Ridge Institute in 2023 (8). Medical schools were selected from the top and from the middle quintile of the Blue Ridge list (see Methods).

All schools in the top quintile had NIH-supported MSTP programs (and no non–NIH-supported MDPhD programs), whereas the middle quintile contained schools that had either MSTP or MDPhD programs. None of the schools in the 4th and 5th quintiles had MSTP programs. The presence of an NIH-funded MSTP or a non–NIH-funded MDPhD program was guided by AAMC designations (9). One school with an unfunded MDPhD program obtained NIH funding during the period under analysis. For that school the student categories for each year corresponded to the funding source for that year.

Only institutions that publicly reported detailed residency match lists for both their combined degree (MSTP or non-MSTP) programs and for their MD-only cohorts were included in the analysis. This criterion yielded 13 schools in the top quintile and 8 schools in the middle quintile. The complete MD match list was purged of those in the MSTP or non-MSTP MDPhD programs to generate the MD-only lists for each institution.

## Results

We conducted this analysis both at the composite student level and at the school level. Matches were analyzed separately for the years 2021, 2022, and 2023 and as an average over those 3 years. A multilevel model was generated for the analysis of statistical outcomes adjusted for covariates.

The rank of the residency match was calculated specifically for every residency match for each Blue Ridge–listed specialty in the year of the match. Two rankings were determined, one as a percentile (e.g., if the residency was the 8th most funded out of 50 listed for that specialty in 2023, a student matching that year would be in the 16th percentile). Average match percentiles by medical school were calculated by averaging the percentiles of every matched residency for every MD, MSTP, or MDPhD student at that school in the match year.

Separately, we calculated the fraction of MD, MSTP, or MDPhD students at each school for each year who matched into the top-10th-percentile residencies in their specialties and averaged these matches for each medical school (e.g., if 30 out of 200 MD-only students at the medical school matched to top-10th-percentile residencies, the fraction would be 0.15).

We only analyzed medical schools that had published lists of individual student matches, both for all medical students and for MSTP or MDPhD students. This yielded 13 medical schools (all with MSTPs) from the top quintile of NIH–funded medical schools and 8 schools (3 with MSTPs and 5 with MDPhD programs) from the middle quintile of Blue Ridge–funded medical schools. The full MD match lists from each school were purged of the MSTP of MDPhD students before analysis of MD-only student matches.

Table 1 shows the number of student matches grouped by year and medical school funding quintile for those matches that were listed to an academic center (i.e., in the specialty-specific Blue Ridge list). Ninety-four percent of publicly listed matches were to academic centers for which NIH funding to the matched specialty could be ascertained from the Blue Ridge “Clinical Science Department” rankings. Table 2 shows the number of student matches similarly grouped for the 6% of students listed as matching to hospitals, for which available funding data were only at the hospital level and the funding rank of the specific matched specialty was unavailable.

Below, we analyze the student matches listed to academic centers and clinical specialties separately from the student matches listed to hospitals. This is based on the assumption that funding of the specialty department is more closely aligned to research resources available to residents than might arise from total NIH funding at the hospital level. Total NIH funding to hospitals (as per Blue Ridge hospital listings) cannot account for differential funding at the specialty level. The exception was for students matched to pediatric hospitals. Because they are single-specialty, we interpolated the funding level of those hospitals into the Blue Ridge Clinical Science Department rankings for Pediatrics.

### Student matches by training background.

We first discuss the match of the 94% of students for whom clinical specialty funding levels at their academic (rather than hospital) matches were listed. For purposes of this analysis, references to NIH funding of residencies indicates the NIH funding rank of the specialty department in which the residency is housed.

**Figure 1:**
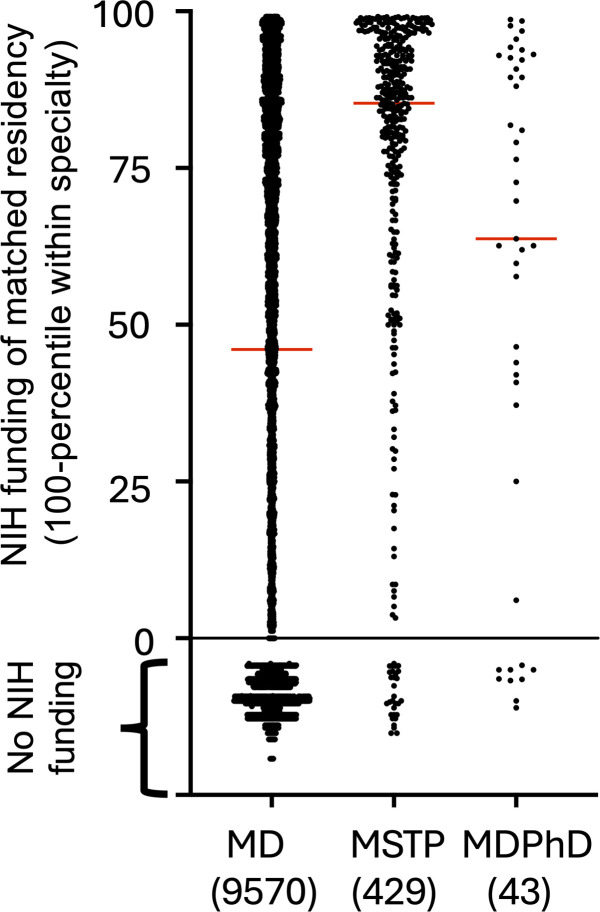
Residency matches of individual students, 2021–2023, by medical school training program. Each dot is a student in the indicated training program (total students/program in parentheses). The *y* axis represents the NIH funding level of each student’s matched residency program within the matched specialty. Higher values indicate more NIH funding within the matched specialty department (e.g., if the residency that a student matched into was in the 20th percentile of funding for that specialty, then the NIH funding is shown as 100 – percentile = 80). Students who matched to programs that did not make the Blue Ridge Institute listing for funding for the specialty in that match year are denoted as “No NIH funding” (see text). The red bar indicates the median for all student matches. For MSTP versus MD, *P* < 0.0001; for MDPhD versus MD, *P* < 0.0001; and for MSTP versus MDPhD, *P* = 0.59, using a linear mixed effects model with percentile as a continuous variable and year adjustment (see Table 3).

In Figure 1, the funding level of residency matches is shown for the 10,022 MD, MSTP, or MDPhD students who had public listings of the academic center and specialty to which they matched. The median percentile matched by students differed by their training program while in medical school, with medians of 54th (95% CI 54th–56th), 15th (95% CI 13th–16th), and 36th (95% CI 12th–56th) percentiles for MD-only, MSTP, and MDPhD, respectively. In Figure 1, the student matches are shown as 100 minus the percentile such that higher values in the figure are aligned with higher funding levels for consistency with other figures.

### Matches of the students at top- versus mid-quintile medical schools.

**Figure 2:**
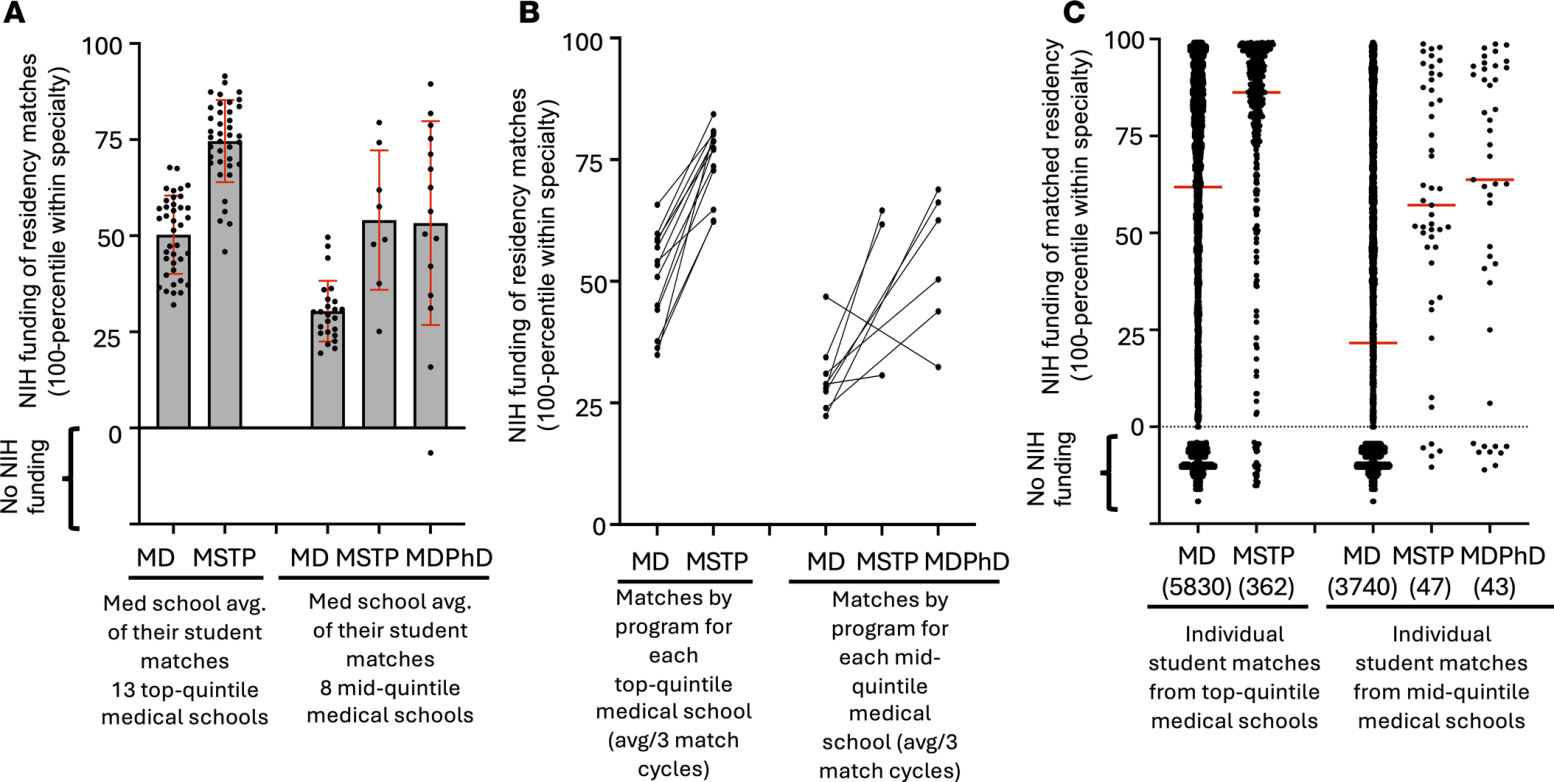
Matches by medical school and training background to residencies ranked by residency NIH funding. (**A**) Each point represents a single medical school’s average match by its students in the indicated groups in a given match cycle (2021–2023). Bars depict the mean NIH funding rank across all of the schools ± standard deviation. The *y* axis value is the average NIH funding level of all residency matches within the school for the indicated training group. It is shown as 100 minus the percentile of each matched residency program within the matched specialty, so that higher values indicate that the school has residency matches in more highly funded specialty departments (e.g., a value of 75 indicates that on average the students in that medical school matched to specialties at schools that were ranked in the 25th percentile for NIH funding). (**B**) Intraschool matches of students in different training programs averaged over 3 match cycles. Each point is a medical school and lines depict the average match of students in the indicated training programs within each school. The *y* axis represents NIH funding as in **A**. (**C**) Each dot is a student in the indicated training program (total students/program in parentheses). The *y* axis represents the NIH funding level of each student’s matched residency program within the matched specialty. As in Figure 1A, higher values indicate more NIH funding within the matched specialty department. Results are grouped by the NIH funding level (by quintile) of the medica l school in which students trained. The red bar shows the median. Significance between training program and between quintiles using a linear mixed effects model with percentile as a continuous variable and year adjustment are shown in Table 3.

Because the level of research resources and NIH support of the medical school at which students train could influence their match, we compared student matches based on the quintile of NIH funding of the trainees’ medical schools. Figure 2A compares medical schools for 3 match cycles, separating top- and mid-quintile schools and the training background of the students at each school. In this representation, each dot is the average at the school in a single match cycle of the residencies to which their students matched. Bars represent an average across schools and cycles. The top quintile of funded medical schools’ MD students aggregated over each match cycle matched to 50th percentile (95% CI 46th–53rd) residencies, and MSTPs to 25th percentile (95% CI 22nd–29th). For mid-quintile medical schools, MD students matched to 70th percentile (95% CI 66th–73rd) residencies, while MSTPs matched to 46th percentile (95% CI 31st–61st) and MDPhDs matched to 47th percentile (95% CI 32nd–61st) residencies. Differences in match rates were sustained after adjustment for year, so this relationship was not driven by outlier years (see also Supplemental Figure 1; supplemental material available online with this article; https://doi.org/10.1172/jci.insight.202606DS1). As in Figure 1, the *y* axis is 100 minus the percentile so that higher funding aligns with higher values.

Figure 2B averages the residency matches for each school over the 3 years and highlights how trainees in different groups within that school performed in the match. Figure 2C shows the distribution of individual student matches (each dot represents a student rather than a school) for each quintile and training program from 2021 to 2023.

The differences between the training program and between the medical school funding quintile were highly significant, as shown in Table 3.

Individual schools are compared by the funding of academic department residency matches of their students in Supplemental Figure 2.

### Matches listed to hospitals.

Six percent of published student matches listed hospitals for which the distribution of NIH funding to specialties within the hospital system cannot be ascertained by the Blue Ridge listing. For these students, we assumed the hospital unit to be the indicator of research resources shaping the residency environment and ranked matches by total hospital NIH funding. Because only 6 students in total had hospitals listed in their match in the mid-quintile MSTP and MDPhD programs over these cycles, that group is not included in the figure.

**Figure 3:**
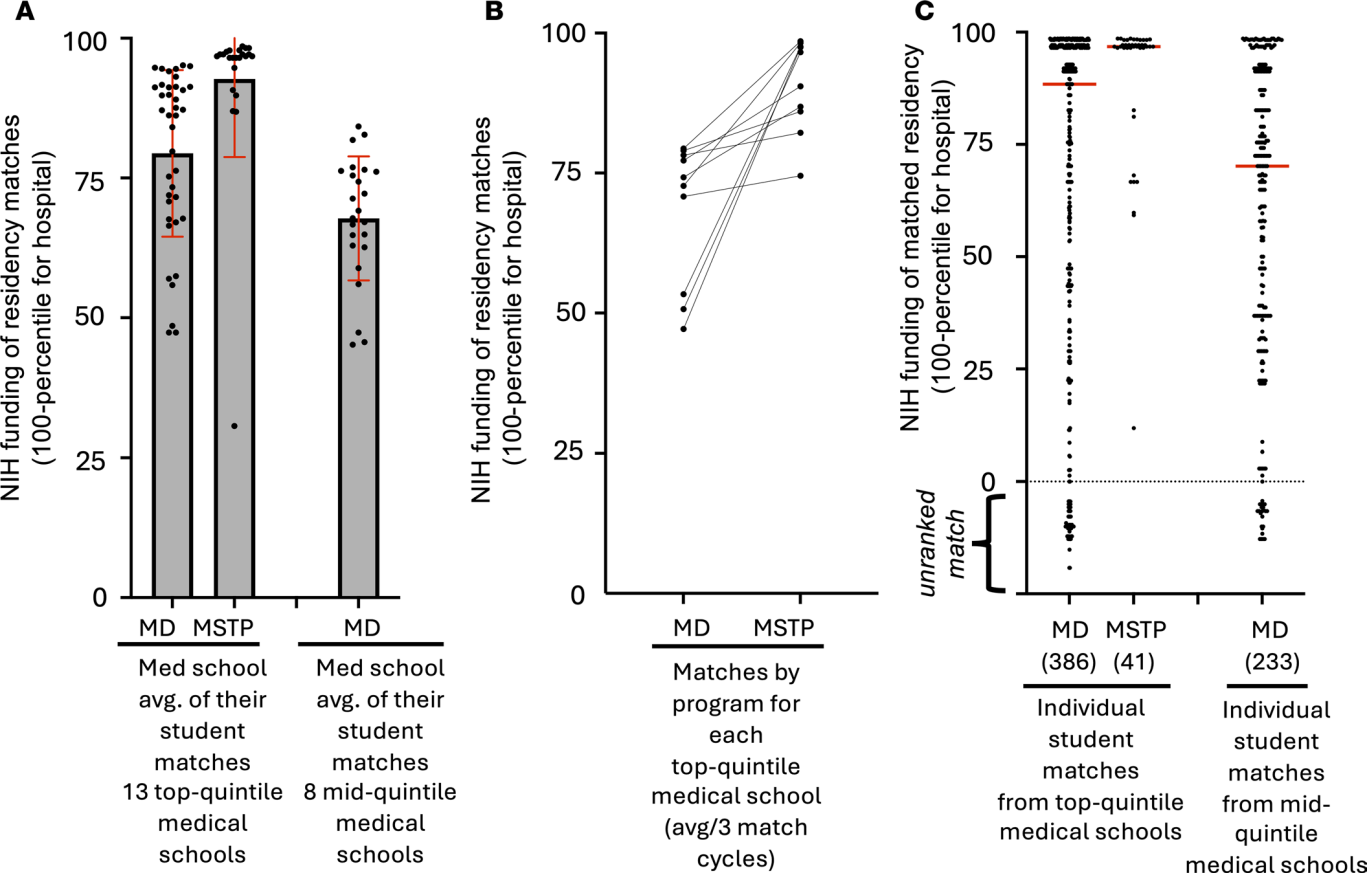
Matches by medical school and training background to residencies at hospitals ranked by residency hospital NIH funding. (**A**) Each dot is a match cycle for a school with average value derived from its matches in 2021, 2022, and 2023 for the indicated groups. The *y* axis represents NIH funding of school’s averages of hospital NIH funding rank matched by its students listed with hospital matches. Funding is shown as 100 minus the percentile (higher is better). (**B**) Matches averaged over 3 cycles shown for each upper-quintile school comparing within-school matches of MD and MSTP students. (**C**) Each dot is a student in the indicated training program (total students/program in parentheses). The *y* axis represents the NIH funding level of each student’s matched hospital. Higher values indicate more NIH funding of the matched hospital. The red bar shows the median. Significance is *P* < 0.0001 for MSTP versus MD in top-quintile using a linear mixed effects model with percentile as a continuous variable and year adjustment.

A similar relationship between training program and matches match level was seen as for departments in Figure 2, with MSTP students in the top quintile matching to higher-NIH-funded residencies than MD students. The top quintile of funded medical schools’ MD students over each cycle averaged to 21st percentile hospitals (95% CI 16th–25th), while MSTPs matched to 7th percentile (95% CI 1st–13th). MD students from 3rd-quintile schools matched to 32nd percentile (95% CI 27th–36th). Figure 3A shows 3 match cycles per institution averaged for student matches and Figure 3B shows the school values averaged over 3 cycles as in Figure 2B. Considering either the school (Figure 3A) or the individual student level (Figure 3C) as units, top-quintile MD-only students matched to higher-funded hospitals than mid-quintile MD-only students.

While multiple factors guide students’ match selections, the most research-guided MSTP and MDPhD students are likely to prioritize the very highest research-supported residency sites in their match list. We therefore analyzed what percentage of the MD-only, MSTP, and MDPhD student body at each school matched to the top-10%-funded residency in their specialty and match year.

**Figure 4:**
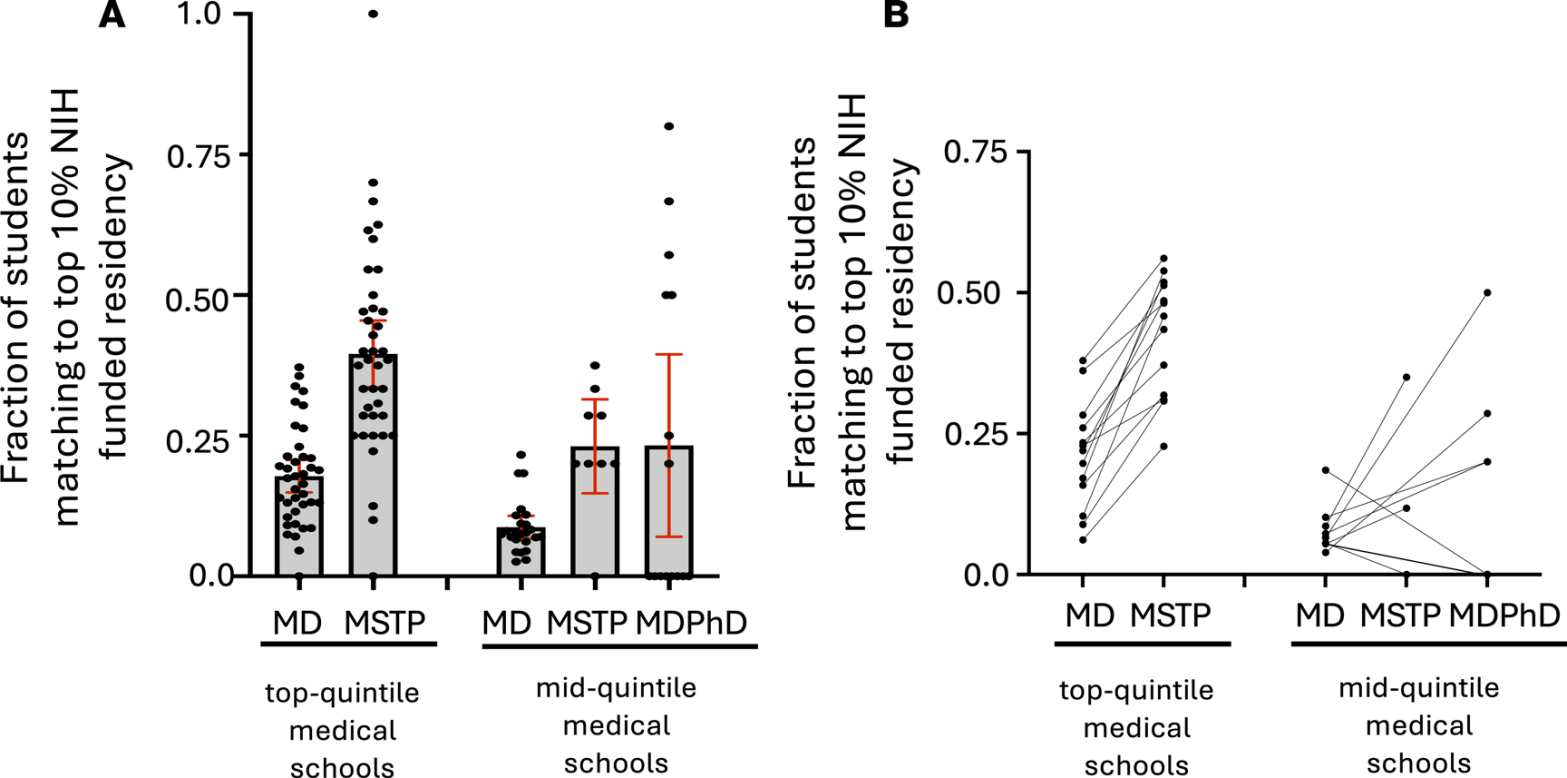
Matches by medical school and training background to top 10% of residencies. (**A**) Each dot is a medical school with the fraction of its students who matched to top-10th-percentile NIH-funded residencies denoted for each of 3 match cycles. The bars represent the average fraction matching at this level across all of the medical schools during each of 3 match cycles. Standard deviation is shown as red bars. Results are divided by training level and by the funding quintile of the medical school that the students attended. (**B**) Each dot represents a single school’s fraction of students matching to top-10th-percentile NIH-funded residencies, averaged over 3 match cycles (2021–2023) for each school. The lines connects each medical school’s outcome for the indicated training groups. Significance between training program and between quintiles were modeled based on a binary outcome of acceptance to the top 10% of funded residencies or not and is shown in Table 4.

Figure 4 shows the percentage of students in medical schools over each 2021–2023 match cycle who matched to the top-10%-funded residency. For both upper and middle quintiles, schools averaged more than twice as many MSTP students matching to the top 10% of residencies as MD-only students using Blue Ridge specialty-specific rankings for the match year. Within-school comparisons showed a higher percentage of MSTPs (or MDPhDs) than MD students matching to top-10%-funded residency for every top-quintile medical school and for most of the mid-quintile schools. Table 4 lists the statistical outcomes for the binary comparison by school and training program of matching to the top-10%-funded residency or not.

## Discussion

This study demonstrates that students trained in dual-degree programs matched to significantly higher-NIH-supported residency departments than MD-only students both across medical schools and within medical schools. We also show that higher NIH funding of medical schools is associated with student matches to higher-NIH-funded residency departments, regardless of training path. While the bulk of our dual-degree data pertained to MSTP programs, the limited MDPhD (i.e., non–NIH-supported) program data that were publically available showed concordant outcomes with MSTP students.

The compatibility of pregraduate and postgraduate research resources of MSTP students’ training and matched residency sites has not been systematically examined previously. Many postgraduate initiatives embody the hope that continuing in a research-rich environment throughout career transitions can sustain the engagement and success of physician-scientists (10, 11).

This report used Blue Ridge Institute listings of medical school and departmental NIH support as a measure to examine this. The match of MD-only students from the same institution as MSTP students served as a control for each match year. We also conducted a comparison of the matching pattern of MSTP students to non–NIH-supported MDPhD students from similarly NIH-funded medical schools.

## Residency matches of MD-only, MSTP, or non-NIH MDPhD trainees

In comparison with MD-only students, MSTP students were disproportionately matched to residencies in departments that had higher NIH funding in the year of the student’s match. Both in aggregate over the 3 years analyzed and in each annual match cycle, both MSTP students and students in MDPhD programs not supported by NIH matched to residency programs within higher-NIH-funded specialty departments. Several analyses showed the same result. These included analyzing the median student match by training program within the entire cohort, analyzing matches within the cohort separated (by quintile) into the NIH funding of their medical school, analyzing the fraction of students matching to top-10%-funded residencies, comparing average matches by training program within medical schools, and comparing average matches by training at all studied schools.

We realize that the difference between MD-only and MSTP/MDPhD students in matching to residencies with high departmental NIH funding may be aligned with different training goals of MD-only versus MDPhD applicants. Matching to a program with the prestige of high NIH funding may not be the option that best fits an applicant’s goals. This metric is not relevant to those seeking matches that will prepare them best for a strictly clinical career. However, an inability of MSTPs to match for residencies in research-engaged departments and institutions could impede their progression into a physician-scientist career.

Of course, MSTP or MDPhD status is not a unidirectional factor impacting what residencies a trainee selects; it is likely to impact residency acceptance decisions as well. Training as an MSTP (or non-NIH MDPhD) could impact residency committee deliberations, particularly for residency specialty sites replete in NIH funding.

We chose to compare matches on the basis of NIH funding of matched residency departments rather than how students at schools matched to research residencies for several reasons. Postgraduate physician-scientist career development programs (e.g., PSTPs) or NIH R38–supported programs in several disciplines do provide a range of training support, including mentorship committees, research resources, and a cohort of research-focused training colleagues (12, 13).

However, using PSTP/R38 program or similar matches as a binary measure to rank resource-rich residency matches is problematic for several reasons: (a) Many available match lists do not specify whether the match was to a research residency track or not. Single-institution reports suggest that one-third of MSTPs match to PSTPs but published data are sparse, and the public match listings fall far short of this number, with less than 0.7% of match listings that we analyzed mentioning research residencies. (b) The content, structure, and time of entry into PSTPs vary, making this a more heterogeneous measure than NIH funding. (c) Departmental NIH funding levels provide a more quantitative, continuous variable for tracking match outcomes of these cohorts.

## Impact of medical school NIH funding on MD-only, MSTP, or non-NIH MDPhD trainee matches

The impact of medical school rank on residency matches has recently been reported for orthopedics matches (14). Linkage of medical school rank and residencies has been noted for other competitive specialties when controlled for in-house matches (15). These papers generally confirmed a correlation of higher medical school status with the stature of the matched residency. However, in both studies, medical school rank was derived from US News and World Report listings, not NIH funding. Doximity (14) or competitiveness of the matched specialty (15) by acceptance rate (16) were used to rank residencies.

To our knowledge, no previous report has compared the pre- and postgraduate affiliations of trainees as a function of NIH funding at the medical school and residency specialty level. While the NRMP has reported superior match rates for many specialties from higher-NIH-funded medical schools (chart 13 in ref. 16), there was no consideration of NIH funding of the matched residency as in this report. Moreover, we believe the current report is the first to compare outcomes within and between schools for MD-only and dual-degree (MSTP and non–NIH-supported MDPhD) trainees. Furthermore, to our knowledge, our single institution report (17) had been the only within-institution comparison of MSTP and non-MSTP matches by residency department funding.

### Medical school ranks and matches by specialty level funding.

Students at top-quintile schools had more matches on average to high-NIH-funded residencies than those at mid-quintile schools, regardless of training program. The difference in the matches of mid-quintile versus top-quintile schools could have many reasons. These may be based on reputation or resource related. Differing medical school culture could also shape the match. Even though the selected mid-quintile schools had an MSTP/MDPhD program, the overall mission of the schools could have focused primarily on excellence in clinical careers.

### Medical school ranks and student matches to hospitals by Blue Ridge–listed NIH funding.

For roughly 6% of the students, matches were listed to hospitals. As in the case of matches listed to academic departments, when medical schools were grouped into quintiles, the MSTP students from top-quintile medical schools matched at significantly higher rates to top-funded hospitals than MD-only students from those schools. Top quintile medical schools outperformed mid-quintile medical schools for MD-only student matches, as in the case for matches to academic departments. We were underpowered for comparisons of the 3 mid-quintile medical schools with MSTP or MDPhD programs for which hospital matches were listed for trainees and did not include them in the analysis.

## Limitations

This study has several limitations. A known limitation arises from using NIH funding rankings from the Blue Ridge Institute as the arbiter of residency research rank (see Methods). The Blue Ridge Institute does not rank all clinical specialties and therefore some student matches to unranked specialties were excluded (see Methods). Moreover, funding at some institutions is linked to hospitals rather than directly to the clinical specialty departments, and therefore the departments are not credited with this money and are listed low in the Blue Ridge listings or are unlisted. In most cases, this is ameliorated because the public match list gives hospital listings that align with those funding arrangements (e.g., matches to Harvard-affiliated hospitals rather than Harvard clinical specialties). The Blue Ridge rankings for hospitals does not break down NIH support to most hospitals by specialty; therefore, the 6% of medical students whose match was listed to hospitals could not have the NIH funding of their residency environment disaggregated into the specialty of their match. Our choice to use total hospital funding to rank matches in situations where listed matches were to hospitals assumes proportionate NIH funding of all matched specialties in the hospitals. Even though this assumption is imperfect, ranking hospital matches in this way gave similar results to the more specific academic clinical department listings.

We recognize that NIH funding of medical schools could be a surrogate marker correlated with reputation or other factors that impact residency admission decisions. The 13 top-quintile medical schools with accessible data had 4 times the average funding of the mid-quintile medical schools ($288M vs. $71M). NIH funding levels could impact reputational factors in the match.

Among the MD applicants to the match, we did not break out medical students in specialized MD-only research track programs that have been implemented in several institutions. Indeed, in a previous study of residency matches at our institution, medical students in our MD-only PSTP research track matched to top-funded residency departments at rates comparable to MSTP students (17). In the current study, the effect of enrollment in such special MD-only programs would be masked by the much greater number of MD-only participants outside of those special tracks.

Although our analysis did not normalize institutional comparisons by the number of students in each track within the institution, this is addressed in part by our comparisons between MD and MSTP students within each institution. Still, for between-quintile comparisons, the relative student sizes between institutions could change how much impact an individual student’s match had on the school’s average. Supplemental Figure 2 shows average outcomes and standard deviations for individual schools in the study to address this.

We analyzed match cycles over 3 years, 2021–2023, for practical reasons. We recognize that COVID-19 affected the medical school training of applicants during these years and might have impacted applications to residency programs in hospitals emerging from crisis phase, particularly in 2021.

Notably, however, there was little difference in the average match outcomes of the medical school’s students when each year was considered separately (Supplemental Figure 1) and our conclusions remained robust after adjustment of *P* values for year.

The residency match is an early measure of how students may sustain a similar research environment in an early postgraduate transition. It is uncertain how well the residency/fellowship research environment predicts long-term research engagement. Variations in program support, mentorship, and geographic residency preference likely contribute to outcomes beyond match rank and funding alone. It would be of interest to know the long-term outcomes for MSTPs in categorical versus research-track postgraduate programs as a comparator to this study.

There is a need for well-controlled studies on the long-term career impact of research residencies. To our knowledge, only one published study has surveyed residents and fellows on their intent to remain in research (18). A low response limits the generalizability of that study, which captured feedback from 94 residents and fellows nationwide. Of those, 65% residents envisioned continuing in research with a 75% time commitment (ref. 18 and Jennifer Kwan, Yale University, personal communication).

The consistent placement of MSTP and MDPhD students in higher-funded residencies and hospitals over multiple match cycles indicates that their medical school training supports a continued trajectory in the physician-scientist pathway. As biomedical research grows more complex, sustaining and expanding these training pathways will be critical to supporting academic medicine and translational science nationwide.

## Methods

The Blue Ridge ranking (“clinical specialties”) of the clinical department housing residency specialty matches was identified for each student for the year of their match. A relatively small number of students were listed as matching to hospitals rather than to departments listed in the Blue Ridge ranking. Hospital research funding differs from institutional funding, as the listing of funding reflects allocation to the hospital as a whole without specification by specialty. The unavailability of specialty-specific data could distort comparisons between students with listed matches into hospitals versus departments at academic institutions. To address this, students who matched into hospitals were filtered out and analyzed separately. This separation was made possible by the Blue Ridge Institute providing 2 distinct datasets, where one listed institutional research funding by specialty and another listed hospital funding.

Pediatric hospitals were excluded from the hospital-specific analysis and instead integrated into the institutional analysis for pediatrics. This is because pediatric hospitals are specialty-specific, with all research funding directed exclusively to pediatrics. For example, if a pediatric hospital were allocated $100 million in funding and the top-ranked pediatric academic institution received $95 million, then the hospital would be ranked first and the institution second in a composite pediatric funding list. Rankings continued in this manner for each year analyzed. Pediatric hospitals were therefore from the hospital rankings dataset to maintain consistency.

Certain specialty-focused hospitals, such as Massachusetts Eye and Ear Infirmary, were retained in the hospital analysis rather than integrated into institutional rankings because their funding spans multiple specialties (e.g., ophthalmology and otolaryngology). The overall effect of breaking hospitals out into a separate dataset was small because only 6% of students were listed to match into hospital-based programs compared with 94% who matched into institution-based programs with specialty-level funding data.

Matches ascribed to clinical specialties were handled according to Blue Ridge NIH funding listings for the specialty for that year. Since Blue Ridge does not rank plastic surgery or vascular surgery, these specialties were grouped under general surgery; interventional radiology was mapped to radiology. For students who matched to combined residencies, e.g., Med-Peds, we used the higher funded of the two disciplines at their matched institutions to calculate match rank, except that Child Neurology was considered a Neurology match. Radiation oncology and maxillofacial surgery matches that lack a near homolog in the Blue Ridge list were not considered in the analysis. Students who only matched into transitional year programs or went directly into the workforce rather than matching into a residency were also excluded from the final dataset and are not counted among the students analyzed. To address matches in specialties ranked by Blue Ridge when the matched institution did not make it onto the Blue Ridge list, an adjustment was applied: 5 points were added to the total program count as a relative-ranked position. For example, if a specialty had 60 ranked programs, then an unranked program in that specialty was assigned a rank of 65. We applied this 5-point “penalty,” as matches without any NIH dollars were qualitatively different in their mission that research-engaged institutions.

The caliber of the research match was analyzed in 2 ways. First, the relative percentile of the matching department was calculated for each student match. For instance, if a student matched into the 20th-most-funded internal medicine department in 2022 (i.e., University of Michigan) out of the 114 listings that year in medicine, then the relative rank of their match would be 20/114 = 0.175 (i.e., 17.5th percentile). Any match to a department not in the Blue Ridge list was given a position 5 below the lowest listed position for that specialty (i.e., 114 + 5 = 119 for an unlisted department of medicine in 2022).

We also calculated how many students matched to the top 10% of departments in their specialty in their match year. For instance, Internal Medicine in 2023 had 113 schools with NIH funding listed by Blue Ridge. Any student matching to one of the top 11 most funded Internal Medicine departments in 2023 would have been considered to have a top 10% residency match.

### Statistics.

We used multilevel mixed-effect logit models to account for students clustering within each school. In each model, the unadjusted and adjusted (for year) *P* values were calculated using the bootstrap method with an unstructured covariance structure.

### Study approval.

This study was determined by the University of Pittsburgh Institutional Review Board to not be human subjects research.

### Data availability.

Values for all data points in graphs are reported in the Supporting Data Values file.

## Author contributions

All authors participated in the conceptualization and implementation of this study. RAS designed this study and contributed to writing, analysis, and figure generation. SB collected all data, conducted analysis and contributed to writing and figure generation. SMN contributed to writing and performed statistical analyses. SB and SMN share first authorship given the discrete essential data gathering and analysis by SB and statistical model generation and analysis by SMN. Author order is alphabetical. All authors reviewed and approved submission of this manuscript.

## Conflict of interest

The authors have declared that no conflicts of interest exist.

## Funding support

University of Pittsburgh School of Medicine institutional support to the University of Pittsburgh/Carnegie Mellon University MSTP program.

## Supplementary Material

Supplemental data

Supporting data values

## Figures and Tables

**Table 1:**
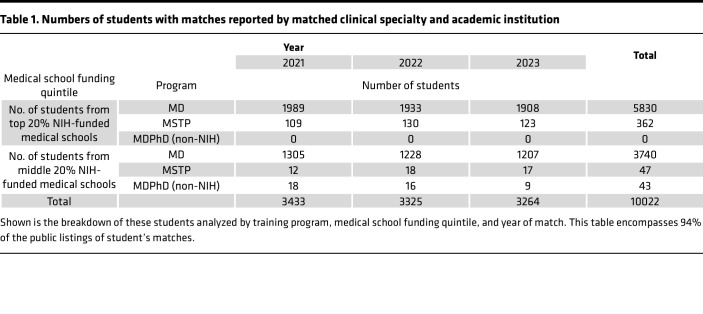
Numbers of students with matches reported by matched clinical specialty and academic institution

**Table 2:**
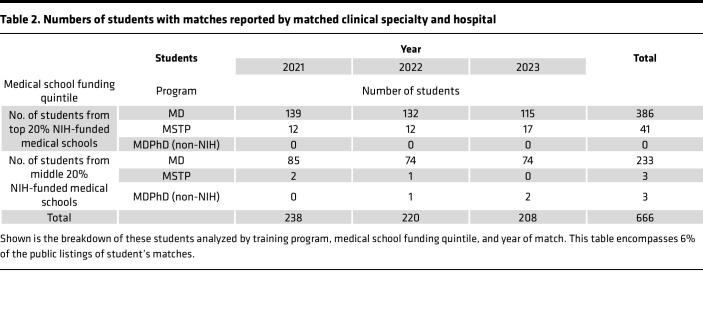
Numbers of students with matches reported by matched clinical specialty and hospital

**Table 3:**
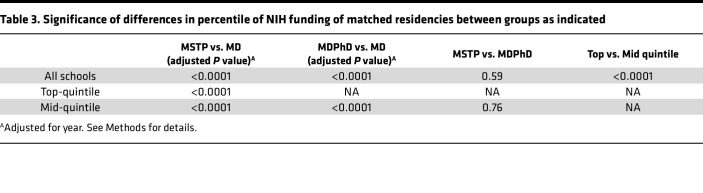
Significance of differences in percentile of NIH funding of matched residencies between groups as indicated

**Table 4:**
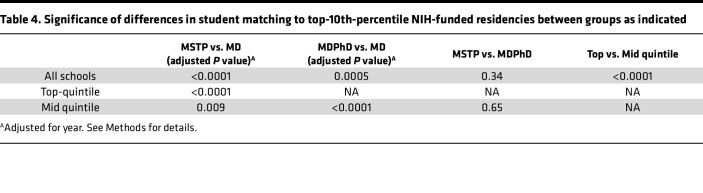
Significance of differences in student matching to top-10th-percentile NIH-funded residencies between groups as indicated

## Version 1 06/22/2026

Electronic publication
