# Supplementary material for: Residency matches of MD-PhD versus MD-only students: compatibility with continued research momentum
Source: JCI Insight. 2026 Jun 22;11(12):e202606. doi: 10.1172/jci.insight.202606 (PMC13313499; doi:10.1172/jci.insight.202606)
Supplement: Supplemental data [file jciinsight-11-202606-s013.pdf]

Table S1. Public listings of student residency matches, 2021-3. The listings used in the study follow. Differences in listings of the same matched Academic Center were resolved manually for mapping to Blue Ridge Institute ranks.

INSTITUTION
2021FlagshipPioneeringFellow
AbbottNorthwesternHosp-MN
AbrazoHealthNetwork-AZ
AdventHealthFlorida
AdventistHealthUkiahValley-CA
AdventistHealthWhiteMemorial
AdventistHealthWhiteMemorial-CA
AdvocateHealthCare
AdvocateHealthCare-IL
AdvocateHealthCare-IL/IllinoisMasonicMedCtr
AdvocateHealthCare-OakLawn,Ill.
AdvocateHealthCare-IL
AdvocateHealthCare–ALGH,ParkRidge,IL
AdvocateHealthCareIL
AIstartup
AkronGeneralMedicalCenter/NEOMED
AkronGenMedCtr/NEOMED-OH
AlamedaHealthSys-HighlandHosp-CA
AlamedaHealthSystem-HighlandHospital
AlamedaHealthSystem-HighlandHospital,CA
AlamedaHealthSystemHighlandHospital
AlbanyMedCtr-NY
AlbanyMedicalCenter
AlbanyMedicalCenter-Albany,N.Y.
AlbanyMedicalCollege
AlbertEinstein
AlbertEinstein/MontefioreMedCtr
AlbertEinsteinCOM
AlbertEinsteinHealthcareNetwork-PA
AlbertEinsteinHealthcareNetwork-PA/Montgomery
AlbertEinsteinHealthcareNetwork-PA
AlleghenyGeneralHospital
AlleghenyGeneralHospital-Pittsburgh,PA
AlleghenyGeneralHospital-Pittsburgh,Pa.
AlleghenyGenHosp-PA

AllinaHealth
AllinaHealth-MN
AllinaHealth-MN
AltruHealthSystem-ND
AmitaHealth,Chicago,IL
AmitaResurrectionMedCtr-IL
AmitaResurrectionMedicalCenter,Chicago,IL
AmitaStFrancisHosp-IL
AnMedHealth
ArizonaHealthScienceCenter
Army-TriplerArmyMedicalCenter
ArnotOgdenMedCtr-NY
ArrowheadRegMedCtr-CA
ArrowhedRegionalMedicalCenter
AscensionProvidence/MichiganStateUniversity
AscensionProvidence/MSUCHM-MI
AscensionProvidence/MSUCollegeofHumanMedicine,MI
AscensionResurrection-IL
AscensionSaintThomasHospitalMidtown
AscensionStJohnHosp-MI
AscensionStVincentHospCtr-IN
AtlantaMedCtrGA
AtlantaMedCtrGA,Atlanta
AtlantaMedStr-GA
AtriumHealthCabarrus-NC
AtriumHealthNavicentMedCtrGA
AuroraStLukesMedicalCtr-WI
AuroraStLukesMedicalCtr-WI
AUUGAMedicalPartnershipGA
AventuraHospital
AventuraHospital-FL
BaptistHealth-AR
BaptistHealth-AR
BaptistHospofSoutheastTexas,Beaumont,TX
BaptistOutreachServices
Barnes-JewishHosp-MO
Barnes-JewishHosp-Mo.
Barnes-JewishHosp-MO(PrimaryCare)
Barnes-JewishHospital
Barnes-JewishHospital-St.Louis,MO

Barnes-JewishHospital-StLouis,MO
Barnes-JewishHospital,MO
Barnes-JewishHospital,St.Louis,MO
Barnes-JewishHospital
BarnesJewishHosp,St.Louis,MO
BarnesJewishHospital
BarnesJewishHospital,St.Louis,MO
BarnesJewishHospital(WashingtonUniversityinSt.Louis)
BarnesJewishHospMO
BarrowNeurologicalInstatSJHMC-AZ
BarrowNeurologicalInstitute
BarrowNeurologicalInstituteatSt.Joseph'sHospital&MedicalCenter
BassettMedicalCenter-Cooperstown,N.Y.
BassettMedicalCenter-Cooperstown,NY
BayCareHealthSystem,Fla.
BayfrontMedCtr-FL
BayfrontMedicalCenter-St.Petersburg,Fla.
BayhealthMedicalCenter
Baylor
Baylor College of Medicine, Houston, TX
Baylor/TexasChildren'sHospital
BaylorCollegeofHouston,Houston,TX
BaylorCollegeofMedicine
BaylorCollegeofMedicine-Houston
BaylorCollegeofMedicine,Houston,TX
BaylorCollMed-Houston-TX
BaylorCollMed-Houston-TX(PSTP)
BaylorCollMed-Houston-TX(ScientistDevelopmentProgram)
BaylorCollMed-TX
BaylorCollMed-Houston-TX
BaylorCollMed,Houston
BaylorCollMedHoustonTX
BaylorCollMedTX
BaylorCOM
BaylorCOM,Houston
BaylorCOM,Houston(Preliminary-InternalMedicine,BaylorCOM,Houston)
BaylorCOM,Houston(TransitionalYear,HCAHoustonHealthcare/UHouston)
BaylorS&WAllSaintsMedCtr-TX
BaylorS&WAllSaintsMedCtr,FortWorth,TX
BaylorS&WMedCtr-Round

BaylorScott&WhiteMedCtr-TX
BaylorScott&WhiteMedCtr,RoundRock,TX
BaylorScott&WhiteMedCtr,Temple,TX
BaylorScott&WhiteMedCtr,Texas
BaylorScott&WhiteMedicalCenter,Temple,TX
BaylorScottandWhiteMedicalCenter
BaylorUMedCtr,Dallas
BaylorUniv
BaylorUnivCoM
BaylorUniversity
BaylorUniversityMedicalCenter
BaylorUniversityMedicalCenter,Dallas,TX
BaylorUnivMedCtr-Dallas-TX
BaylorUnivMedCtrDallasTX
BaystateFranklinMedCtr-MA
BaystateMedCtr-MA
BaystateMedicalCenter
BaystateMedicalCenter-Springfield,MA
BaystateMedicalCenter,Springfield,MA
BeaumontHealth
BeaumontHealth-MI
BeaumontHealth-MI(RoyalOak)
BeaumontHealth-MI(Wayne)
BeaumontHealth,MI
BethesdaHospital-OH
BethIsraelDeaconessMedCtr-MA
BethIsraelDeaconessMedCtr,Mass.
BethIsraelDeaconessMedCtr.,Mass.
BethIsraelDeaconessMedicalCenter
BethIsraelDeaconessMedicalCenter-Boston,MA
BethIsraelDeaconessMedicalCenter-Boston,Mass.
BethIsraelDeaconessMedicalCenter,Boston,MA
BethIsraelDeaconessMedicalCenter,MA
BethIsraelDeaconessMedicalCtr,Boston,MA
BIDeaconessMedCtr-MA
BIDeaconessMedCtr-MA(PrimaryCare)
BIDeaconessMedCtrMA
BIDeaconessMedicalCenter
BIDeaconessMedicalCenter–Boston,MA
BIDeaconessMedicalCenter–Brockton,MA

BillingsClinic-MT
BiotechIndustry
BoiseVAMedCtr-ID
BonSecoursHealthSystem
BonSecoursHealthSystem-VA
BostonChildren's
BostonChildren'sHospital
BostonChildren’sHospital
BostonChildren’sHospital-Boston,MA
BostonCombinedProgram
BostonMedicalCenter–Boston,MA
BostonUniversity
BostonUniversityMedicalCenter
BostonUniversityMedicalCenter-Boston,MA
BostonUniversityMedicalCenter–Boston,MA
BostonUnivMedCtr
BostonUnivMedCtr-MA
BostonUnivMedCtr-MA
BostonUnivMedCtr,Boston
BostonUnivMedCtrMA
Brigham&Women's
Brigham&Women's/MassGeneral
Brigham&Women'sHosp
Brigham&Women’sHosp
Brigham&Women'sHosp-MA
Brigham&Women’sHospital
Brigham&Women’sHospital-Boston,MA
Brigham&Women’sHospital–Boston,MA
Brigham&Women’sHospital,Boston,MA
Brigham&Women’sHospital,MA
Brigham&Women'sHospital(Harvard)
Brigham&Women'sHospital/MassGeneral
Brigham&WomensHosp
Brigham&WomensHosp-MA
Brigham&WomensHosp-MA/BWH-MGH
Brigham&WomensHosp-MA
Brigham&WomensHospital
Brigham&WomensHospital/Harvard
Brigham&WomensHospMA
BrighamandWomen’sHosp,Mass.

BrighamandWomen'sHospital
BrighamandWomen'sHospital(Child)
BrookeArmyMedicalCenter
BrookeArmyMedicalCenter-SanAntonio,TX
BrooklynHospCtr-NY
BrookwoodBaptistHealth
BrookwoodBaptistHealth,Ala.
BrookwoodBaptistHealth,Birmingham,AL
BrowardHealthMedCtrFL
BrowardHealthMedicalCenter
BrownU/RhodeIslandHosp
BrownU/Women&InfantsHospofR.I.
BrownUniv/ButlerHospital-RI
BrownUniv/KentHospital
BrownUniv/RhodeIslandHosp
BrownUniv/RhodeIslandHosp,Providence,RI
BrownUniv/Women&InfantsHospofRI
BrownUniversity
BrownUniversity/ButlerHospital
BrownUniversity/ButlerHospital-Providence,RI
BrownUniversity/KentHospital
BrownUniversity/RhodeIslandHospital
BrownUniversity/RhodeIslandHospital-Providence,R.I.
BrownUniversity/RhodeIslandHospital-Providence,RI
BrownUniversity/RhodeIslandHospital–Providence,RI
BrownUniversity/RhodeIslandHospital,Providence,RI
BrownUniversity/Women&InfantsHospitalofRI
CahabaMedicalCare
CahabaMedicalCare-AL
CahabaMedicalCareAL
CaliforniaPacificMedicalCenter
CaliforniaPacificMedicalCenter-SanFrancisco,CA
CambridgeHealthAlliance-Cambridge,MA
CambridgeHealthAlliance,Mass.
CambridgeHealthAllianceCambridgeHospital
CampbellUniversity
CampbellUniversity-NC
CampbellUniversity,Fayetteville,NC
CampPendleton
CAPacificMedCenter

CAPacificMedicalCenter
CAPacificMedicalCenter,SanFrancisco
CapitalHealthRegionalMedicalCenter-Pennington,NJ
CarilionClinic-VATech
CarilionClinic-VirginiaTechCarilionSOM
CarilionClinic-VirginiaTechCarilionSOM
CarleFoundationHosp-IL
CarlR.DarnallArmyMedCtr-Ft.Hood,Killeen,TX
CarlR.DarnallArmyMedicalCenter
CarlR.DarnallArmyMedicalCenter,FortHood,TX
CarlRDarnallArmyMedicalCenter,FortHood,TX
CarolinasMedCtr
CarolinasMedCtr-NC
CarolinasMedCtr-NC
CarolinasMedCtr,Charlotte
CarolinasMedCtrNC
CarolinasMedicalCenter
CarolinasMedicalCenter-Charlotte,NC
CarolinasMedicalCenterProgram/AtriumHealth
CarolinasMedicalCtr
CasaColinaHosp-CA
CasaColinaHospital
CaseWestern
CaseWestern/MetroHealthMedCtr-OH
CaseWestern/MetroHealthMedicalCenter
CaseWestern/MetroHealthMedicalCenter-Cleveland,OH
CaseWestern/UHospsClevelandMC,Cleveland,OH
CaseWestern/UHospsClevelandMedCtr,Cleveland,OH
CaseWestern/UHospsClevelandMedCtr,Ohio
CaseWestern/UniversityHospitalsClevelandMedical
CaseWestern/UnivHosps
CaseWestern/UnivHospsCaseMedCtr-OH
CaseWestern/UnivHospsClevelandMedCtr
CaseWestern/UnivHospsClevelandMedCtr-OH
CaseWestern/UnivHospsClevelandMedCtr,Cleveland,OH
CaseWestern/UnivHospsClevelandMedCtrOH
CaseWesternReserveUniversity
CaseWesternReserveUniversity-Cleveland,OH
CaseWesternReserveUniversity/UniversityHospitalsClevelandMedicalCenter
CaseWesternUniveristyHospitalsClevelandMedicalCenter

CaseWesternUniversityMedicalCenter,Cleveland,OH
Cedars-Sinai
Cedars-SinaiMedCtr
Cedars-SinaiMedCtr-CA
Cedars-SinaiMedCtr,Calif.
Cedars-SinaiMedicalCenter
Cedars-SinaiMedicalCenter-LosAngeles,CA
Cedars-SinaiMedicalCenter,LosAngeles,CA
Cedars-SinaiMedicalCtr
Cedars-SinaiMedCenter
CedarsSinai
CedarsSinaiMedicalCenter
CenterforFamilyMedicine-SD
CentralIowaHealthSystem
CentralMichiganUniversityCOM
CentralWAFamilyMed
CenturaHealth/HealthCampus,Westminister,CO
CharlesR.DrewU,Calif.
CharlesR.DrewUniversity
CharlesRDrewUniversity-CA
Chattanooga
Children’sHosp-Boston
Children’sHosp,Boston
Children’sHosp,Philadelphia
Children'sHospital-Boston
Children’sHospital-Boston
Children'sHospital-Boston,MA
Children'sHospital-LA
Children’sHospital-LA,LosAngeles,CA
Children'sHospital-LosAngeles
Children'sHospital-Oakland
Children'sHospital-Philadelphia
Children’sHospital-Philadelphia,Philadelphia,PA
Children'sHospital-Philadelphia
Children’sHospital,Boston
Children’sHospital,Boston,MA
Children’sHospital,LosAngeles
Children’sHospital,Oakland,CA
Children'sHospital,Philadelphia,PA
Children’sHospitalBoston–Boston,MA

Children'sHospitalLosAngeles
Children'sHospitalMedicalCenter
Children’sHospitalofBoston
Children'sHospitalofMichigan
Children'sHospitalofPhiladelphia
Children’sHospitalofPhiladelphia
Children'sHospitalofPhiladelphia-Philadelphia,Pa.
Children'sHospitalofPittsburgh
Children'sMedicalCenter
Children’sMercyHospital,KansasCity,KS
Children'sNationalHospital
Children'sNationalMedicalCenter
Childrens+T41:T60Hospital-Boston-MA
ChildrensHosp-Philadelphia-PA
ChildrensHosp-Philedelphia
ChildrensHosp-Philadelphia-PA
ChildrensHospital-Boston-MA
ChildrensHospital-LA-CA
ChildrensHospital-NEOMED-OH
ChildrensHospital-Oakland-CA
ChildrensHospital-Boston-MA
ChildrensHospital-Oakland-CA
ChildrensHospitalBostonMA
ChildrensHospitalLACA
ChildrensHospitalOaklandCA
ChildrensHospitalofBoston
ChildrensHospitalofPhiladelphia
ChildrensHospitalofPhiladelphia-Philadelphia,PA
ChildrensHospPhiladelphiaPA
ChildrensMercyHospital-MO
ChildrensNationalMedCtr-DC
ChildrensNationalMedCtr-DC
ChildrensNationalMedicalCenter-Washington,DC
ChristHospital
ChristHospital-OH
ChristianaCare
ChristianaCare-DE
ChristianaCare-DE
ChristianaCare–Newark,DE
ChristianaCare,DE

ChristianaCare,Del.
ChristianaCareDE
CHRISTUSHealth-GSMC,Longview,TX
CHRISTUSHealth-SantaRosa,SanAntonio,TX
CHRISTUSHealth–SantaRosa,SanAntonio,TX
CHRISTUSHealth–Spohn,CorpusChristi,TX
CHRISTUSHealth,Texas
CincinnatiChildren'sHospital
CincinnatiChildren’sHospital-Cincinnati,OH
CincinnatiChildren'sHospitalMedicalCenter
CincinnatiChildren’sHospitalMedicalCenter
CincinnatiChildren'sHospitalMedicalCenter-Cincinnati,Ohio
CincinnatiChildren’sHospitalMedicalCenter,OH
CincinnatiChildren’sHospMedCtr
CincinnatiChildren’sMedicalCenter
CincinnatiChildrensHospital
CincinnatiChildrensHospitalMedicalCenter-Cincinnati,OH
CincinnatiChildrensHospitalMedicalCenter,Cincinnati,Ohio
CincinnatiChildrensHospMedCtr-OH
CincinnatiChildrensHospMedCtrOH
CityofHope
CityofHope-CA
ClevelandClinic
ClevelandClinic-ColeEyeInstitute
ClevelandClinicFdn
ClevelandClinicFdn-OH
ClevelandClinicFdn-OH
ClevelandClinicFdn,Ohio
ClevelandClinicFlorida
ClevelandClinicFoundation
ClevelandClinicFoundation-Cleveland,OH
ClevelandClinicFoundation-Cleveland,Ohio
ClevelandClinicFoundation–Cleveland,OH
ClevelandClinicFoundation,Cleveland,OH
CMSRU/CooperUniversityHospital-NJ
CMSRU/CooperUnivHospital,Camden,NJ
ColiseumMedCtrs
ColiseumMedCtrs-GA
CollegeofHumanMedicine
ColumbiaUniv

ColumbiaUniv/StamfordHospital
ColumbiaUniversity/NewYork-PresbyterianHos
ColumbiaUniversity/NewYork-PresbyterianHospital
ColumbiaUniversityCollegeofPhysiciansandSurgeons
CommunityHealthCare-WA
ConeHealth
ConemaughMemorialMedicalCenter
ConroeRegionalMedicalCenter,Conroe,TX
ContraCostaRegionalMedicalCenter
ContraCostaRegionalMedicalCenter-Martinez,CA
ContraCostaRegionalMedicalCenter,Martinez,CA
ContraCostaRegMedCtr-CA
CookCounty-StrogerHospital-IL
CookCountyHealthandHospitalsSystem
CookCountyHealthandHospsSys-IL
CookCountyHealthandHospsSys-IL
CookCountyHealthandHospSystem,Ill.
CooperMedicalSchoolofRowanUniversity
CooperMedicalSchoolofRowanUniversity/CooperUniversityHospital-Camden,N.J.
CornellUniv
CornellUniversity
CPMC-SanFrancisco
CreedmoorPsychCtr-NY
CreightonUnivAffilHosps-AZ
CreightonUnivAffilHosps-NE
CreightonUniversity
CreightonUniversity-AZ
CreightonUniversity-AZ/Phoenix
CreightonUniversity-NE
CreightonUniversity-AZ
CreightonUniversity-NE
CreightonUniversity,Neb.
CreightonUniversity,Omaha,NE
CreightonUniversitySchoolofMedicine-ValleywiseHealthMedicalCenter
Crozer-ChesterMedCtr-PA
Crozer-ChesterMedicalCenter
Ctr-GAEmoryUnivSOM-GA
CWRU/MetroHealthMedicalCenter
CWRU/UniversityHospitalsClevelandMedicalCenter
DanburyHospital-Danbury,CT

DarnallArmyMedicalCenter
DarnallArmyMedicalCenteratFortHood,TX
Dartmouth-HitchcockMedCtr-NH
Dartmouth-HitchcockMedCtr,Lebanon,NH
Dartmouth-HitchcockMedicalCenter
Dartmouth-HitchcockMedicalCenter-Lebanon,NH
Dartmouth-HitchcockMedicalCenter–Lebanon,NH
Dartmouth-HitchcockMedicalCenter,Lebanon,NH
Dartmouth-HitchcockMedicalCenter,NH
Dartmouth-HitchcockMedCtr-NH
DartmouthHitchcockMedCtr,Lebanon,NH
DartmouthHitchcockMedCtrNH
DartmouthHitchcockMedicalCenter-Lebanon,NH
DenverHealthMedCtr-CO
DenverHealthMedicalCenter
DenverHealthMedicalCenter-Denver,CO
DenverHealthMedicalCenter,Denver,CO
DeTarHealthcareSystem,Victoria,TX
DetroitMedCtr/WSU-MI
DetroitMedCtr/WSU-MI-Harper/VA
DetroitMedCtr/WSU-MI
DetroitMedCtr/WSU,Mich.
DetroitMedicalCenter
DetroitMedicalCenter/WayneStateUniversity
DetroitMedicalCenter/WSU,Detroit,MI
DignityMethodistHosp-Sacramento-CA
DignityMethodistHospital-Sacramento
Drexel/StChristophersHospforChildren
Duke
DukeUMedCtr,N.C.
DukeUMedCtr.,N.C.
DukeUniv
DukeUniversity
DukeUniversityMedCtr,Durham,NC
DukeUniversityMedicalCenter
DukeUniversityMedicalCenter-Durham,NC
DukeUniversityMedicalCenter-NC
DukeUniversityMedicalCenter–Durham,NC
DukeUniversityMedicalCenter,Durham,NC
DukeUniversityMedicalCenter,NC

DukeUniversityMedicalCenter(Research)
DukeUniversityMedicalCtr,Durham,NC
DukeUnivMedCtr
DukeUnivMedCtr-NC
DukeUnivMedCtr-NC
DukeUnivMedCtr,Durham,NC
DukeUnivMedCtr,N.C.
DukeUnivMedCtrNC
DukeUnivMedicalCenter-Durham
DwightDavidEisenhowerArmyMedicalCenter
EastAlabamaMedicalCenter
EasternCTHealthNetwork
EasternIdahoRegionalMedCtr,IdahoFalls,ID
EasternIdahoRegionalMedicalCenter
EasternIdahoRegMedCtr
EasternVAMedSchool-VA
EasternVirginiaMedicalSchool
EasternVirginiaMedicalSchool-Norfolk,VA
EasternVirginiaMedicalSchool-Norfolk,Va.
EasternVirginiaMedSchool
EasternVirginiaMedSchool,Norfolk,VA
EastFloridaGMEConsortium,Plantation,FL
EastFloridaGraduateMedicalEducationConsortium-Plantation,Fla.
EastJeffersonGenHosp-LA
EastJeffersonGenHosp-LA
EastTennesseeStateUniversity
EastTennesseeStUniv
ECUHealthMedCtr-NC
Einstein
Einstein/MontefioreMedCtr-NY
EisenhowerArmyMedicalCenter
EisenhowerMedCtr-CA
ElsonSFloydCOMWSU-WA
EmanateHealth-CA
EmoryUniv
EmoryUniversity
EmoryUniversityHospital
EmoryUniversitySchoolofMedicine
EmoryUniversitySchoolofMedicine-Atlanta,GA
EmoryUniversitySchoolofMedicine,Atlanta,GA

EmoryUniversitySchoolofMedicine,Atlanta,Georgia
EmoryUniversitySchoolofMedicine,GA
EmoryUniversitySchoolofMedicineAtlanta-GA
EmoryUniversitySOM
EmoryUnivSchoolofMedicine-Atlanta,GA
EmoryUnivSoM
EmoryUnivSOM-GA
EmoryUnivSOM-GA
EmoryUnivSOM,Ga.
EmoryUnivSOMGA
EmoryUnivSOMGA,Atlanta,GA
EmoryUSOM,Ga.
EmoryUSOM,Ga.(Preliminary-InternalMedicine,UTMedBranch,Galveston)
EmoryUSOM,Ga.(TransitionalYear,EmoryUSOM,Ga.)
FamilyHealthCtrsatNYULangone-NY
FamilyHealthCtrsatNYULangoneNY
FamilyHealthCtrsofSanDiego-CA
FamilyMedicineResofIdaho
FAU-SchmidtCollegeofMedicine,BocaRaton,FL
FAUSchmidt-COM-FL
FloridaAtlanticUniversity-SchmidtCollegeofMedicine-BocaRaton,Fla.
FloridaHosp-Orlando-FL
FloydMedCtrGA
FortBelvoirCommunityHospital
FortBelvoirCommunityHospital-FortBelvoir,VA
FortBenningMartinArmyCommunityHospital
FranciscanHealth-IN
FullCircleHealth-ID
FullCircleHealth–Boise,ID
GardenCityHospital-Detroit,MI
GatewayBehavioralHealth
GatewayBehavioralHlthCommSvc-GA
GeisingerHealth
GeisingerHealthSystem-Bloomsburg,Pa.
GeisingerHealthSystem-Danville,PA
GeisingerHealthSystem-Danville,Pa.
GeisingerHealthSystem-PA
GeorgetownUniv
GeorgetownUniversity/WashingtonHospital
GeorgetownUnivHosp-DC

GeorgeWashington/ChildrensNational
GeorgeWashingtonUniv
GeorgeWashingtonUniv-DC
GeorgeWashingtonUniv-DC
GeorgeWashingtonUniversity
GeorgeWashingtonUniversity-Washington,DC
GeorgeWashingtonUniversity,Washington,DC
GeorgeWashingtonUniversity/Children'sNationalMedicalCenter
GeorgeWashingtonUniversityHospital
GeorgeWashU,D.C.
GoodSamaritanHosp-Cinn-OH
GoodSamaritanHospital
Google
GrandRapidsMedEdPartners-MI
GrandStrandRegMedCtr
GrandStrandRegMedCtr-SC
GrandStrandRegMedCtrSC
GreaterBaltimoreMedicalCenter,Baltimore
GreaterLawrenceFamilyHealthCenter
GreaterLawrenceFamilyHealthCenter-Lawrence,MA
GreaterLawrenceFamilyHealthCenter–Lawrence,MA
GreaterLawrenceFamilyHlthCtr,Lawrence,MA
GreenwichHospital-CT
GrtrLawrenceFamHlthCtr-MA
GrtrLawrenceFamHlthCtr-MA
GundersenLutheranMedFdn-WI
GundersenLutheranMedFdn-WI
Guthrie/RobertPackerHosp-PA
HalifaxMedCtr-FL
HalifaxMedicalCenter
Harbor-UCLAMedCtr
Harbor-UCLAMedCtr-CA
Harbor-UCLAMedCtr,Calif.(TransitionalYear,HCAHoustonHealthcare/UHouston)
Harbor-UCLAMedCtr.
Harbor-UCLAMedicalCenter
Harbor-UCLAMedicalCenter,Torrance,CA
Harbor-UCLAMedicalCenter,WestCarson,CA
Harbor-UCLAMedCtr-CA
Harbor-UCLAMedicalCenter
Harvard/BethIsraelDeaconessMedCtr

Harvard/BIDMC
Harvard/BostonChildren’s
Harvard/BostonChildrensHospital
Harvard/Brigham&WomensHosp
Harvard/BWH
Harvard/CambridgeHealthAlliance
Harvard/MassachusettsGeneralHosp
Harvard/MassEyeandEar
Harvard/MassGeneralHosp
Harvard/MGH
Harvard/Spaulding-MA
HarvardMedicalSchool-Mass.Eye&EarInfirmary
HarvardMedicalSchool/MassachusettsEyeandEar(Research)
HarvardMedSchool-Ear&EyeInfirmary
HarvardMedSchool/MassEyeandEar
HarvardSouthShore
HarvardSouthShore-Brockton,MA
HarvardSouthShore-MA
HarvardSouthShore,Brockton,MA
HarvardSpauldingRehabHosp-MA
HarvardSpauldingRehabHosp,Charlestown,MA
HarvardSpauldingRehabHospMA
HarvardSpauldingRehabilitationHospital
HarvardSpauldingRehabilitationHospital–Charlestown,MA
HarvardUniversity/BostonChildren'sHospital
HarvardUniversity/Brigham&Women'sHospital
HarvardUniversity/Children'sHospital
HarvardUniversity/MassachusettsGeneralHospital
HCA Houston Hlthcare/U Houston-West, Houston, TX
HCABrandonRegional
HCAHealthcare/TriStar,Nashville,TN
HCAHealthcare/TriStarNashville-TN
HCAHealthcare/TriStarNashville-TN
HCAHealthcare/TriStarSouthernHills-TN
HCAHealthcare/UniversityofSouthFloridaMorsaniCollof
HCAHealthcare/USFMorsaniBayonetPtFL
HCAHealthcare/USFMorsaniGME-Citrus-FL
HCAHealthcare/USFMorsaniGME-OakHill,Fla.
HCAHealthcare/USFMorsaniGME-Trinity-FL
HCAHealthcareEastFLDivisionGME

HCAHealthcareLGH-Montgomery/VCOM-VA
HCAHoustonHealthcare-West,Houston,TX
HCAHoustonHealthcare/UHouston
HCAHoustonHealthcare/UHouston-TX
HCAHoustonHealthcare/UHouston-TX
HCAHoustonHealthcare/UniversityofHouston-Houston,Texas
HCAHoustonHlthcare/UHouston-West,Houston,TX
HCAHoustonHlthcare/UHouston,Kingwood,TX
HCAHoustonHlthcare/UofHouston,Houston,TX
HCALasPalmasdelSolHealthcare-TX
HCALasPalmasdelSolHealthcare,ElPaso,TX
HCAMedicalCityHealthcare
HCAMedicalCityHealthcare-TX
HCAMedicalCityHealthcare,Arlington,TX
HCAMedicalCityHealthcare,FortWorth,TX
HCAMedicalCityHealthcare,Texas
HCAMedicalCityHealthcare,Weatherford,TX
HCAMedicalCityHlthcare,Weatherford,TX
HealthEducationServices-TN
HealthONE–AuroraMedCtr,LoneTree,CO
HealthONE–SkyRidgeMedCtr,LoneTree,CO
HealthONE–SwedishMedCtr,Englewood,CO
HealthONE,Colo.
HealthPartnersInstitute-MN
HealthPartnersInstitute-MN
HealthPartnersInstitute,MN
HennepinCoMedCtr-MN
HennepinCoMedCtr-MN
HenryFordHospital
HenryFordHospital-MI
HenryFordHospital-MI|HenryFordHospital-MI
HenryFordHospital,Detroit
HenryFordHospital,Detroit,MI
HenryFordHospital,MI
HenryFordHospitalMI
HenryFordHSC-MI
HenryFordMacombHospital-MI
HenryFordWyandotteHospital,Wyandotte,MI
HersheyMedCtr/PennState-PA
HiloMedicalCenter-HI

HofstraNorthwellSchofMed,NewHydePark,NY
HofstraNorthwellSOM
HonorHealth-AZ
HospForSpecialSurgery-NY
HospforSpecialSurgery,N.Y.
Hospital
HospitalForSpecialSurgery
HospitaloftheUniversityofPA
HospitaloftheUniversityofPennsylvania
HospitaloftheUniversityofPennsylvania-Philadelphia,PA
HospitaloftheUniversityofPennsylvania,Philadelphia
HospitaloftheUnivofPenn
HospitaloftheUofPennsylvania,Philadelphia,PA
HospoftheUnivofPA
HospoftheUnivofPA(PrimaryCare)
HospoftheUnivofPennsylvania,Philadelphia,PA
HospoftheUofPA
HospoftheUofPa.
HospoftheUofPennsylvania,Philadelphia,PA
HospUMichiganHosps-AnnArbor
HoustonMethodist
HoustonMethodistHosp
HoustonMethodistHospital
HowardUnivHosp-DC
HunterdonMedicalCenter-Flemington,N.J.
HuntingtonHealth
HuntingtonMemorialHosp-CA
HuntingtonMemorialHospital
HuntingtonMemorialHospital-Pasadena,Calif.
Icahn-MountSinai
IcahnSchoolofMedicineatMorningsideWest
IcahnSchoolofMedicineatMountSinai
IcahnSchoolofMedicineatMountSinai-MountSinaiBethIsrael-NewYork,N.Y.
IcahnSchoolofMedicineatMountSinaiHospital-NewYork,NY
IcahnSchoolofMedicineatMountSinaiHospital-NYC,NY
IcahnSchoolofMedicineatMountSinaiHospital,NewYork,NY
IcahnSchoolofMedicineatMountSinaiHospital,NY
IcahnSchoolofMedicineatMountSinaiMorningside-West-NewYork,NY
IcahnSchoolofMedicineatMtSinai
IcahnSOMatMountSinai

IcahnSOMatMountSinaiHospital,NewYork,NY
IchanSchoolofMedicineatMountSinaiMorningsideWest
IdahoStateUniversity
IllinoisEyeandEarInfirmary
IllinoisEyeandEarInfirmary,UniversityofIllinois–Chicago,IL
IndianaUniversity
IndianaUniversityMedicalCenter
IndianaUniversitySchofMed,Indianapolis,IN
IndianaUniversitySchoolofMedicine
IndianaUniversitySchoolofMedicine-Indianapolis,IN
IndianaUniversitySchoolofMedicine,Indianapolis,IN
IndianaUniversitySOM
IndianaUniversitySOM,Indianapolis,IN
IndianaUniversitySOM(Indianapolis)
IndianaUnivHealthMedCtr
IndianaUnivSchOfMed
IndianaUnivSchoolofMedicine,Indianapolis,IN
IndianaUnivSoM
IndianaUSOM
IndianaUSOM,Ga.
Industry
InHisImageFamilyMed,Tulsa,Okla.
InovaFairfaxHospital
InovaFairfaxHospital-FallsChurch,Va.
InovaFairfaxHospital-VA
InovaFairfaxHospital,VA
Insitituion
InspiraHealthNetwork-MullicaHill,NJ
InstituteforFamilyHealth
InstituteforFamilyHealth-NY
InstituteforFamilyHealth,NY
IntermountainMedCtr-UT
ISMMSMountSinaiBethIsrael-NewYork,NY
ISMMSMountSinaiBethIsrael-NY
ISMMSMountSinaiBethIsrael,NewYork,NY
ISMMSMountSinaiHosp,N.Y.
ISMMSMountSinaiHospital
ISMMSMountSinaiHospital-NewYork,NY
ISMMSMountSinaiHospital-NY(PrimaryCare)
ISMMSMountSinaiHospital-NY

ISMMSMountSinaiHospital,N.Y.
ISMMSMountSinaiHospital,NewYork,NY
ISMMSMountSinaiMorningside-West
ISMMSMountSinaiMorningside-West-NY
ISMMSMountSinaiMorningside-West,NewYork,NY
ISMMSMountSinaiMorningside-West-NY
ISMMSMountSinaiSouthNassau-NY
J.P.Morgan–NewYork,NY
JackHughstonMemorialHospAL
JacksonMemorialHosp-FL
JacobiMedCtr/Einstein-NY
JamaicaHospMedCtr-NY
JeffersonHealth-Northeast
JeffersonHealth-NortheastPA
JeffersonMedColl/duPontChildren’s,Philadelphia,PA
JeffersonMedicalCollege
JeffersonMedicalCollege/duPontHospitalforChildren
JerseyShoreUMedCtr,N.J.
JewishHospital-OH
JohnMuirHealth
JohnMuirHealth-WalnutCreek,CA
JohnPeterSmithHosp-TX
JohnPeterSmithHosp,FortWorth
JohnPeterSmithHosp,Texas
JohnPeterSmithHospital
JohnPeterSmithHospital,FortWorth,TX
JohnsHopkins
JohnsHopkins/Bayview-Baltimore,Md.
JohnsHopkins/Bayview-MD
JohnsHopkins/Bayview-MD|JohnsHopkinsHosp-MD
JohnsHopkinsAllChildrensHosp-FL
JohnsHopkinsAllChildrensHospital
JohnsHopkinsBayview
JohnsHopkinsBayviewMedicalCenter
JohnsHopkinsHosp-MD
JohnsHopkinsHosp-MD
JohnsHopkinsHosp,Baltimore,MD
JohnsHopkinsHosp,Md.
JohnsHopkinsHospital
JohnsHopkinsHospital-Baltimore,MD

JohnsHopkinsHospital-Baltimore,Md.
JohnsHopkinsHospital–Baltimore,MD
JohnsHopkinsHospital,Baltimore,MD
JohnsHopkinsHospital,Baltimore,Md.
JohnsHopkinsHospital,MD
JohnsHopkinsHospMD
JohnsHopkinsMedHospital
JohnsHopkinsUniv
JohnsHopkinsUniversity
JohnsHopkinsUniversity,Baltimore,MD
JohnstonMemorialHospital-VA
KadlecRegionalMedCtr-WA
KaiserPermanente
KaiserPermanente-CapitolHill,Seattle,WA
KaiserPermanente-CentralValley,Modesto,CA
KaiserPermanente-Fontana
KaiserPermanente-LosAngeles
KaiserPermanente-LosAngeles-CA
KaiserPermanente-LosAngeles-LosAngeles,CA
KaiserPermanente-LosAngeles,CA
KaiserPermanente-Oakland
KaiserPermanente-Oakland-CA
KaiserPermanente-Oakland-CA(PrimaryCare/Equity&Disparities)
KaiserPermanente-OrangeCo-CA
KaiserPermanente-Riverside-CA
KaiserPermanente-SanDiego-CA
KaiserPermanente-SanJose
KaiserPermanente-SanJose-CA
KaiserPermanente-SantaClara-CA
KaiserPermanente-SantaRosa-CA
KaiserPermanente-SF-CA
KaiserPermanente-WoodlandHills
KaiserPermanente-WoodlandHills-CA
KaiserPermanente-SanDiego-CA
KaiserPermanente-SanJose-CA
KaiserPermanente–SanJose,CA
KaiserPermanente,SanFrancisco
KaiserPermanenteHawaii
KaiserPermanenteLosAngeles,Pasadena,CA
KaiserPermanenteSanFranciscoMedicalCenter

KaiserPermanenteWashington
KaweahDeltaHealthCareDistrict
KaweahDeltaHealthCareDistrict-CA
KeckMedicine
KeckMedicine-CA/USouthernCalifornia
KeckMedicineofUSC-USCRoskiEyeInstitute
KeeslerAFBMedicalCenter,Biloxi,MS
KendallRegionalMedicalCenter,Miami,FL
KernMedicalCenter
KetteringMedCtr-OH
KirkKerkorianSchoolofMedicine
KirkKerkorianSOMatUniv.ofNevada,LasVegas,NV
KirkKerkorianSOMatUNLV-NV
KirkKerkorianSOMatUNLV-NV
KirkKerkorianSOMatUNLV,Nev.
KootenaiHealth-ID
KootenaiHealth-ID
KPCHealthHemetValleyMedCtr-CA
KresgeEyeInstitute-WayneStateUniversity
KresgeEyeInstitute/WayneStateUniversity
LaheyClinic
LaheyClinic-Burlington,Mass.
LaheyClinic-MA
LaheyClinicMedicalCenter
LakelandRegionalHealth-FL
LancasterGeneralHospital-Lancaster,PA
LancasterGeneralHospital,Lancaster,PA
LancasterGenHosp-PA
LancasterGenHospPA
LankenauMedCtr-PA
LankenauMedicalCenter-Wynnewood,PA
LankenauMedicalCenter-Wynnewood,Pa.
LawndaleChristianHealthCenter-Chicago,IL
LegacyEmanuel/GoodSamaritan-OR
LegacySalmonCreekMedCtr-WA
LehighValleyHosp-PA
LehighValleyHospital
LehighValleyHospital-Allentown,PA
LehighValleyHospital-Allentown,Pa.
LewisGaleMedCtr-VA

LifelongMedicalCare-CA
LincolnMedCtr-NY
LincolnMedicalCenter,Bronx,NY
LincolnMedicalCtr-NY
LomaLindaU,Calif.
LomaLindaUniversity
LomaLindaUniversity-CA
LomaLindaUniversity,LomaLinda,CA
LomaLindaUnivMedCtr-Murrieta-CA
LomaLindaUnivMedicalCenter-Murrieta
LongBeachMemMedCtrCA
LongBeachMemorialMedicalCenter
LosRoblesHealthSystem
LouisianaStateUniversityHealthSciencesCenter
LouisianaStateUniversitySchoolofMedicine-Baton
LouisianaStateUniversitySchoolofMedicine-NewOrleans-NewOrleans,LA
LoyolaUMedCtr,Ill.
LoyolaUniversity
LoyolaUniversity/HinesVAHospital
LoyolaUniversity/StritchSchoolofMedicine
LoyolaUniversityMedicalCenter
LoyolaUniversityMedicalCenter-Maywood,Ill.
LoyolaUnivMedCtr-IL
LoyolaUnivMedCtr-IL
LoyolaUnivMedCtrIL
LSUHealthNewOrleansSchoolofMedicine
LSUHealthScienceCenter,Shreveport,LA
LSUHealthScienceCenterUniversityHospital&Clinics
LSUHlthScienceCenter,Shreveport,LA
LSUHSC-Shreveport-LA
LSUHSC-Shreveport,La.
LSUHSC,Shreveport,La.
LSUHSCUniversityHosps&Clinics-LA
LSUHSCUniversityHosps&Clinics-LA
LSUSchofMed–OLOL,NewOrleans,LA
LSUSchoolofMedicine
LSUSchoolofMedicine,BatonRouge,LA
LSUSchoolofMedicine,NewOrleans,LA
LSUSOM-BatonRouge-LA
LSUSOM-NewOrleans-LA

LSUSOM-NewOrleans,La.
LSUSOM-BatonRouge-LA
LSUSOM-NewOrleans-LA
LSUSOM,NewOrleans
LSUSOMNewOrleansLA
LurieChildren'sHospitalofChicago/McGawMedicalCenterofNorthwesternUniversity
LUTHSC,SanAntonio
LynchburgFamMedRes-VA
MacNealHospital
Madigan
MadiganArmyMedialCenter
MadiganArmyMedicalCenter
MadiganArmyMedicalCenter-LakewoodWA
MadiganArmyMedicalCenter-Tacoma,WA
MadiganArmyMedicalCenteratJointBaseLewis-Mccord
MagnoliaRegionalHealthCenter
MaimonidesMedCtr-NY
MaimonidesMedicalCenter
MaimonidesMedicalCenter-Brooklyn,NY
Maine-DartmouthFamilyMed
MaineMedCtr
MaineMedicalCenter
MaineMedicalCenter-Portand,Maine
MaineMedicalCenter-Portland,Maine
MaineMedicalCenter-Portland,ME
MaineMedicalCenter,Portland,ME
MainlineHealth/BrynMawr
MarianRegionalMedCtr-CA
MarshallUniversitySchofMed,Huntington,WV
MarshallUniversitySOM-WV
MarshallUniversitySOMWV
MarshfieldClinic-WI
MarshfieldClinic-WI
MartinArmyCommunityHospital
MartinLutherKingJr.CommunityHospital
MaryFreeBedHospital-GrandRapids,MI
MassachuettsGenHosp
MassachusettsEye&Ear-HarvardMedicalSchool
MassachusettsEyeandEar(Harvard)
MassachusettsGeneralHosp

MassachusettsGeneralHosp-Harvard,Boston,MA
MassachusettsGeneralHospital
MassachusettsGeneralHospital-Boston,MA
MassachusettsGeneralHospital-Boston,Mass.
MassachusettsGeneralHospital-HarvardCombined
MassachusettsGeneralHospital–Boston,MA
MassachusettsGeneralHospital,Boston
MassachusettsGeneralHospital,Boston,MA
MassachusettsGeneralHospital(Harvard)
MassachusettsGeneralHospital/HarvardCombined,Boston
MassachusettsGenHosp
MassachusettsGenHosp,Boston,MA
MassachusettsGenHosp(PSTP)
MassGeneral
MassGenHosp
MassGenHosp(TransitionalYear,MemorialSloan-Kettering,N.Y.)
Mayo
MayoClinic-Rochester,Mich.nn.
MayoClinicArizona
MayoClinicSchofGradMedEdu,Rochester,MN
MayoClinicSchofGradMedEduc,Jacksonville,FL
MayoClinicSchofGradMedEduc,Rochester,MN
MayoClinicSchoolofGradMedEduc-AZ
MayoClinicSchoolofGradMedEduc-FL
MayoClinicSchoolofGradMedEduc-M
MayoClinicSchoolofGradMedEduc-MN
MayoClinicSchoolofGradMedEduc-AZ
MayoClinicSchoolofGradMedEduc-FL
MayoClinicSchoolofGradMedEduc-MN
MayoClinicSchoolofGradMedEduc-RochesterMN
MayoClinicSchoolofGradMedEduc,Ariz.
MayoClinicSchoolofGradMedEduc,Minn.
MayoClinicSchoolofGradMedEducFL
MayoClinicSchoolofGradMedEducMN
MayoClinicSchoolofGraduateMedEd
MayoClinicSchoolofGraduateMedEduc,Minn.
MayoClinicSchoolofGraduateMedicalEd
MayoClinicSchoolofGraduateMedicalEducation
MayoClinicSchoolofGraduateMedicalEducation-Jacksonville,FL
MayoClinicSchoolofGraduateMedicalEducation-Rochester,MN

MayoClinicSchoolofGraduateMedicalEducation-Rochester,NY
MayoClinicSchoolofGraduateMedicalEducation–Rochester,MN
MayoClinicSchoolofGraduateMedicalEducation,MN
MayoClinicSchoolofGraduateMedicalEducation,Scottsdale,AZ
McGawMedicalCenterofNorthwesternUniversity
McGawMedicalCenterofNorthwesternUniversity-Chicago,IL
McGawMedicalCenterofNorthwesternUniversity,LurieChildren'sHospitalofChicago
McKay-DeeHospitalCtr-UT
McLarenHealthCareCorpMI
McLarenRegMedCtr-MI
McLennanCountyFamMed
MCW
MDAnderson
MedCollWisconsinAffilHosps
MedCtrNavicentHealth/MercerSOM-GA
MedCWisconsin
MedicalCollegeofGeorgia
MedicalCollegeofGeorgia,Augusta
MedicalCollegeofGeorgia,Augusta,GA
MedicalCollegeofWisconsin
MedicalCollegeofWisconsin,Milwaukee,WI
MedicalCollegeofWisconsinAffiliatedHospitals
MedicalCollegeofWisconsinAffiliateHospitals
MedicalUniversityofSC
MedicalUniversityofSC,Charleston,SC
MedicalUniversityofSouthCarolina
MedicalUniversityofSouthCarolina-Charleston,S.C.
MedicalUniversityofSouthCarolina-Charleston,SC
MedicalUniversityofSouthCaroline
MedicalUnivofSouthCarolina,Charleston,SC
MedicialUniversityofSC
Medicine
Medicine-Columbia
MedicineGreenville
MedRegionalMedicalCtrBayonetPoint
MedStarGeorgetownUniversityHospital
MedStarGeorgetownUnivHosp
MedStarGeorgetownUnivHosp-DC
MedStarGeorgetownUnivHosp-DC
MedStarGeorgetownUnivHospDC

MedStarNationalRehabilitationHospital-Washington,DC
MedStarUnionMemorialHosp-MD
MedStarUnionMemorialHospital
MedStarWashingtonHospCtr-DC
MedStarWashingtonHospCtr-DC
MedStarWashingtonHospCtr,Washington,DC
MedStarWashingtonHospitalCenter
MedStarWashingtonHospitalCenter-Washington,D.C.
MedStarWashingtonHospitalCenter-Washington,DC
MedUofSC
Meharry/MetroGeneralHosp
MemorialHealth-UniversityMedicalCenter-Savannah,Ga.
MemorialHealth-UnivMedCtr-GA
MemorialHealthcareSystem-FL
MemorialHealthcareSystem-FL
MemorialHealthcareSystem,Fla
MemorialHermannHosp,Texas
MemorialHermannHospital,Houston,TX
MemorialHermannHospital,SugarLand,TX
MemorialHospital-IN
MemorialSloan-Kettering
MemorialSloan-Kettering-NewYork,N.Y.
MemorialSloan-Kettering-NY
MemorialSloan-Kettering,N.Y.
MemorialSloan-Kettering,NewYork,NY
MemorialSloanKetteringCancerCenter
MercyCatholicMedCtr-PA
MercyHealth-StRitasMedCtr-OH
MercyHealthGrandRapids-GrandRapids,MI
MercyHealthMuskegon
MercyHealthSystem-WI
MercyHospital
MercyHospitalStLouis-MO
MercyMedCtr-DesMoines-IA
MercyMedCtr-DesMoines-IA
MercyOneNorthIowaMedCtr
MeritusMedicalCenter-MD
MethodistHealthSystem,Dallas
MethodistHealthSystem,Dallas,TX
MethodistHealthSystemDallas

MethodistHosp,Houston
MethodistHospital-Houston-TX
MethodistHospital,Houston
MethodistHospital,Houston,TX
MethodistHospitalHoustonTX
MetrohealthMedicalCenter
MichiganStateUniversity-SpectrumHealth
MichiganStUnivCHM-EastLansing
MiddlesexHospital-CT
MiltonS.HersheyMedicalCenter
MissionCommunityHosp-CA
MontanaFamilyMedicine
MontefioreMedCenter/Einstein/Jacobi,Bronx,NY
MontefioreMedCtr/Einstein
MontefioreMedCtr/Einstein-NY
MontefioreMedCtr/Einstein-NY
MontefioreMedCtr/Einstein,N.Y.
MontefioreMedicalCenter
MontefioreMedicalCenter-Einstein,Bronx,NY
MontefioreMedicalCenter,theUniversityHospitalforAlbertEinsteinCollegeofMedicine
MontefioreMedicalCenter/AlbertEinsteinCollegeofMedicine
MontefioreMedicalCenter/Einstein
MontefioreMedicalCenter/Einstein-Bronx,NY
MontefioreMedicalCenter/Einstein–Bronx,NY
MontefioreMedicalCenter/Einstein,Bronx,NY
MontefioreMedicalCenter/Einstein,NY
MontefioreMedicalCenter/EinsteinCampus
MontefioreMedicalCtr,EinsteinHosp,Bronx,NY
MontefioreMedicalCtr/EinsteinHosp,Bronx,NY
MoorehouseSchoolofMedicine
MorehouseSchofMed-GA
MorehouseSchofMedGA
MorehouseSchoolofMedicine,Atlanta,GA
MorehouseSchoolofMedicine,Ga.
MorristownMedCtr-NJ
MorristownMedicalCenter
MountainAHEC
MountainAHEC-NC
MountainAHEC-NC(Asheville)
MountainAreaHealthEducationCenter

MountainAreaHealthEducationCenter-Asheville,NC
MountainViewRegMedCtr-NM
MountCarmelHealthSystem
MountCarmelHealthSystem-OH
MountSinaiBethIsrael,NewYork,NY
MountSinaiHospital
MountSinaiMedicalCenter
MountSinaiMorningside-West
MtAuburnHospital-Boston,MA
MtSinaiMedCenter
MtSinaiMedCtr-Miami-FL
MultiCareGoodSamaritanHosp-WA
MultiCareGoodSamaritanHospital
MultiCareHealthSystem-WA
NassauUniversityMedicalCenter
NassauUnivMedCtr-NY
NationwideChildren'sHosp,Columbus,OH
NationwideChildren'sHospital
NationwideChildrensHosp-OH
NationwideChildrensHospital-Columbus,OH
NavalHospital,Jacksonville,FL
NavalHospitalCampPendleton
NavalMedCtr,Portsmouth,Va.
NavalMedicalCenter
NavalMedicalCenter-Portsmouth
NavalMedicalCenter-Portsmouth,VA
NavalMedicalCenter-SanDiego
NavalMedicalCenter–SanDiego,CA
NavalMedicalCenter,Portsmouth,VA
NavalMedicalCenter,SanDiego
NavalMedicalCenteratSanDiego-SanDiego,Calif.
NavalMedicalCenterCampLejeune
NavalMedicalCenterPortsmouth
NavalMedicalCenterSanDiego
Navy-WalterReedNationalMilitaryMedicalCenter
NavyHospitalJacksonville
NazarethHospital
NazarethHospitalPA
NCCWalterReedNationalMilitaryMedicalCtr
NCCWalterReedNtlMedicalCenter

NemoursChildren'sHospital,Orlando,FL
NemoursChildrensHosp-FL
NemoursChildrensHospFL
NeurologicalSurgery
NewHanoverRegMedCtr
NewHanoverRegMedCtr-NC
NewYork-Presbyterian/Columbia
NewYork-Presbyterian/Cornell
NewYork-Presbyterian/Queens
NewYork-Presbyterian/Queens-Flushing,N.Y.
NewYork-Presbyterian/WeillCornellMedicalCenter
NewYork-PresbyterianHospital-ColumbiaandCornell
NewYork-PresbyterianHospital,ColumbiaUniversityMedicalCenter,NY
NewYork-PresbyterianHospital,CornellMedicalCenter
NewYork-PresbyterianHospital,WeillCornellMedicalCenter,NY
NewYork-PresbyterianHospital/ColumbiaUniversityIrvingMedicalCenter
NewYork-PresbyterianHospital/WeillCornellMedicalCenter
NewYork-PresbyterianBrooklynMethodistHospital
NewYorkEye&EarInfirmary
NewYorkEyeandEarInfirmaryNY
NewYorkMetropolitanHospCtr
NewYorkPresbyterian-ColumbiaUniversity
NewYorkPresbyterianHospital-ColumbiaUniversityMedicalCenter-NewYork,N.Y.
NewYorkPresbyterianHospital-WeillCornell,NewYork,NY
NewYorkPresbyterianHospital-WeillCornellMedicalCenter
NewYorkPresbyterianHospital-WeillCornellMedicalCenter-NYC,NY
NewYorkPresbyterianHospital-Columbia&Cornell
NewYorkPresbyterianHospital-ColumbiaUniversityMedicalCenter
NewYorkPresbyterianHospital-WeillCornellMedCenter
NewYorkPresbyterianHospital-WeillCornellMedicalCenter
NewYorkPresbyterianHospital/Columbia-NewYork,NY
NewYorkPresbyteryHospital-ColumbiaUniversityMedicalCenter-NewYork,NY
NewYorkUniv
NewYorkUniversity
NewYorkUniversityGrossmanSchoolofMedicine-NewYork,N.Y.
NISMMSMountSinaiHospital-NY
NorthColoradoMedCtr
NortheastGeorgiaMedCtr
NortheastGeorgiaMedicalCenter
NorthernNMFamilyMed

NorthShore-LIJHealthSys-NY
NorthsideHospitalGwinnett-GA
NorthsideHospitalGwinnettGA
Northwestern
Northwestern/LurieChildren'sHosp
NorthwesternMcGaw,NMH/VA,Chicago,IL
NorthwesternMcGaw/LurieChildren's
NorthwesternMcGaw/LurieChildren’s
NorthwesternMcGaw/LurieChildren’s,IL
NorthwesternMcGaw/LurieChildrens
NorthwesternMcGaw/LurieChildrens,Ill.
NorthwesternMcGaw/LurieChildrensIL
NorthwesternMcGaw/NMH/VA
NorthwesternMcGaw/NMH/VA-Chicago,IL
NorthwesternMcGaw/NMH/VA-IL
NorthwesternMcGaw/NMH/VA-IL-HumboldtPark
NorthwesternMcGaw/NMH/VA-IL(PSTP)
NorthwesternMcGaw/NMH/VA-IL/LakeForest
NorthwesternMcGaw/NMH/VA,IL
NorthwesternMcGaw/NMH/Va.-Ill.
NorthwesternMcGaw/NMH/Va.-Ill.(Preliminary-InternalMedicine,KaiserPermanente-SanFrancisco)
NorthwesternMcGaw/RIC-IL
NorthwesternMcGaw/RIC,Chicago,IL
NorthwesternMcGaw/ShirleyRyanAbilityLab-IL
NorthwesternMcGraw/NMH/VA-IL
NorthwesternMemorialHospital
NorthwesternMemorialHospital,Chicago,IL
NorthwesternUniversity/LurieChildren'sHospitalofChicago
NorthwesternUniversity/McGawMedicalCenter
NorthwesternUniversityFeinberg
NorthwesternUniversityFeinbergSOM
NovantHealth-NC
NuvanceHealth
NuvanceHealth-NY
NuvanceHealth,Poughkeepsie,NY
NYMC-MetropolitanHospCenter,NewYork,NY
NYMC-MetropolitanHospCtr-NY
NYMC-MetropolitanHospitalCenter
NYPBrooklynMethodistHospNY

NYPHosp-Columbia&Cornell-NY
NYPHosp-Columbia&Cornell,N.Y.
NYPHosp-ColumbiaUMedCtr,N.Y.
NYPHosp-ColumbiaUnivMedCtr
NYPHosp-ColumbiaUnivMedCtr-NY
NYPHosp-ColumbiaUnivMedCtr,NewYork,NY
NYPHosp-WeillCornell
NYPHosp-WeillCornellMedCtr-NY
NYPHosp-WeillCornellMedCtr-NY(PrimaryCare)
NYPHosp-WeillCornellMedCtr,N.Y.
NYPHosp-Columbia&Cornell-NY
NYPHosp-ColumbiaUnivMedCtr-NY
NYPHosp-WeillCornellMedCtr-NY
NYPHosp–ColumbiaUnivMedCtr,NewYork,NY
NYPHospColumbiaUnivMedCtr,NewYork,NY
NYPHospital-ColumbiaMedicalCenter
NYPHospital-ColumbiaUniversityMedicalCenter
NYPHospital-WeillCornellMedicalCenter
NYPHospital-WeillMC/CornellUniversity
NYPHospital–ColumbiaUniversityMedicalCenter–NewYork,NY
NYPHospital–WeillCornellMedicalCenter–NewYork,NY
NYPHospitalColumbia&Cornell
NYPHospitalColumbiaUniversityMedCenter
NYPHospWeillCornellMedCtr,NewYork,NY
NYPresbyterianHosp/CornellMedCtr
NYPresbyterianHospital-WeillCornellMedicalCenter
NYPresbyterianHospitalColumbia&Cornell
NYPresbyterianHospitalWeillMC/CornellU
NYU
NYUGrossmanSchoolofMedicine
NYUGrossmanSchoolOfMedicine-NY
NYUGrossmanSchoolOfMedicine-NY(PrimaryCare)
NYUGrossmanSchoolOfMedicine-NY(PSTP)
NYUGrossmanSchoolOfMedicine-NY
NYUGrossmanSchoolofMedicine–NewYork,NY
NYUGrossmanSchoolofMedicine,NewYork,NY
NYUGrossmanSchoolofMedicine,NY
NYUGrossmanSchoolOfMedicineNY
NYUGrossmanSoM
NYUGrossmanSOM,N.Y.

NYUGrossmanSOM,N.Y.(Preliminary-InternalMedicine,PresbyterianHosp,Dallas)
NYULongIslandSOM-NY
NYUSchoolOfMedicine
NYUSOM
OBHBrookdaleUnivHospMedCtr,Brooklyn,NY
OceanMedCtr-NJ
OchsnerClinicFoundation
OchsnerClinicFoundation-LA
OchsnerClinicFoundation-LA
OchsnerClinicFoundation,La.
OchsnerClinicFoundation,NewOrleans,LA
OchsnerClinicFoundationLA
OchsnerMedicalCenter
OffuttAirForceBase-Omaha,NE
OhioHealth-GrantMedicalCenter-Columbus,OH
OhioHealth-RiversideMethodist
OhioStateUMedCtr
OhioStateUMedCtr(Preliminary-InternalMedicine,MedCollWisconsinAffilHosps)
OhioStateUMedCtr(TransitionalYear,BrookwoodBaptistHealth,Ala.)
OhioStateUniv
OhioStateUniversityMedCtr
OhioStateUniversityMedicalCenter
OhioStateUniversityMedicalCenter-Columbus,Ohio
OhioStateUniversityWexnerMedicalCenter
OhioStateUnivMedicalCenter,Columbus,OH
OHSU
OliveView-UCLAMedCtr-CA
OliveView-UCLAMedicalCenter
OliveView-UCLAMedCtr-CA
OliveView-UCLAMedicalCenter
OliveView–UCLAMedicalCenter,Sylmar,CAUCLAMedicalCenter,LosAngeles,CA
OrangeParkMedicalCenter
OregonHealth&SciencesUniv.
OregonHealth&ScienceU
OregonHealth&ScienceU(Preliminary-InternalMedicine,BaylorScott&WhiteMedCtr,Texas)
OregonHealth&ScienceUniv
OregonHealth&ScienceUniversity
OregonHealth&ScienceUniversity-Portland,OR
OregonHealthandScienceUniversity-Portland,Ore.
OregonHealthSciencesUniv,Portland,OR

OregonHSU
OrlandoHealth
OrlandoHealth-FL
OrlandoHealth-Orlando,FL
OrlandoHealth/OrlandoRegionalMedicalCenter
OrlandoHealthFL
OurLadyoftheLakeRegMedCtr-LA
PalmBeachConsortiumforGME,Fla.
PalmBeachConsortiumforGraduateMedical
PeaceHealthSouthwestMedCtr,Wash.
Pediatrics
PennHighlandsHealthcare-Dubois,Pa.
PennState
PennStateHealthMiltonS.HersheyMedicalCenter
PennStateHealthMiltonS.HersheyMedicalCenter-Hershey,PA
PennStateHealthMiltonS.HersheyMedicalCenter-Hershey,Pa.
PennStateHealthMiltonS.HersheyMedicalCenter-Reading,PA
PennStateHealthSt.JosephMedicalCenter-Reading,Pa.
PennStateHersheyMedCtr-PA
PennStateHersheyMedCtr-PA
PennStateHersheyMedCtr,Hershey,PA
PennStateHersheyMedCtrPA
PennStateHersheyMedicalCenter
PennStateHersheyMedicalCenter-Lancaster,PA
PennsylvaniaHospital
PennsylvaniaHospital-Philadelphia,PA
PennsylvaniaHospital–Philadelphia,PA
PhoebePutneyMemHosp-GA
PhoenixChildren’sHosp,Ariz.
PhoenixChildren'sHospital
PhoenixChildren’sHospital,Phoenix,AX
PhoenixChildren’sHospital,Phoenix,AZ
PhoenixChildrensHospital-AZ
PhoenixChildrensHospital-Phoenix,AZ
PhoenixChildrensHospitalAZ
PiedmontAthensRegional-GA
PiedmontColumbusRegionalGA
PiedmontMaconMedCtr,Macon,GA
PiedmontMaconMedicalCenter
PIHHealthDowneyHospital

PORTSMOUTHNAVALHOSPITAL
PortsmouthRegionalHospital
PortsmouthRegionalHospital-Portsmouth,NH
PoudreValleyHospital
PoudreValleyHospital-CO
PredictionHealth
PresbyterianHosp,Dallas
PresbyterianIntercommunityHospital
PrimeWestConsortium
Prisma
PrismaHealth-UniversityofSouthCarolinaSchoolofMedicine
PrismaHealth-UniversityofSouthCarolinaSchoolofMedicineatColumbia-Columbia,S.C.
PrismaHealth-UniversityofSouthCarolinaSchoolofMedicineGreenville
PrismaHealth-UniversityofSouthCarolineSchoolofMedicineatGreenville-Greenville,S.C.
PrismaHealth-UofSCSOMColumbia
PrismaHealth-UofSCSOMGreenville
PrismaHealth-Upstate/UniversityofSouthCarolinaSchoolof
PrismaHealth-UofSCSOMGreenville
PrismaHealthUniversityofSouthCarolinaSchoolof
PrismaHealthUofSCSOMColumbia
PrismaHealthUofSCSOMColumbia,Columbia,SC
PrismaHealthUofSCSOMGreenville
PrismaHealthUofSCSOMGreenville,Greenville
PrismaHealthUofSCSOMGreenville,Greenville,SC
Program
ProHealthWaukeshaMemorialHospital
ProvidenceHealth
ProvidenceHealth-OR
ProvidenceHealth-OR
ProvidenceHospital-AK
ProvidenceHospital,Anchorage,AK
ProvidenceSacredHeartMedCtr-WA
ProvidenceStPeterHospital-WA
Queen’sUniversity
RapidCityHospital,RapidCity,SD
RapidesRegMedCtr-LA
RedmondMedicalCenter
RedmondRegionalMedCtrGA
RedmondRegionalMedicalCente
RedmondRegionalMedicalCenter-Rome,Ga.

ResidencyInstitution
ResurrectionMedCtr-IL
RichmondUnivMedCtr-NY
RiversideCommunityHospital
RiversideCommunityHospital-CA
RiversideCommunityHospital-Riverside,CA
RiversideCommunityHospital-CA
RiversideCommunityHospital,Riverside,CA
RiversideCountyRegMedCtr-CA
RiversideMethodist-OH
RiversideRegionalMedicalCenter-NewportNews,Va.
RiversideRegionalMedicalCenter–NewportNews,VA
RiversideRegMedCtr-VA
RiversideUniversityHealthSys-CA
RiversideUniversityHealthSystem
RochesterGeneralHospital–Rochester,NY
RochesterGeneralHospital,Rochester,NY
RochesterGenHosp-NY
RochesterGenHosp,N.Y.(TransitionalYear,HCALasPalmasdelSolHealthcare,ElPaso)
Rock
Rock-TX
RogerWilliamsMedCtr-RI
RogerWilliamsMedicalCenter
RogerWilliamsMedicalCenter–Providence,RI
Rouge
Rush-CopleyMemHosp-IL
RushUMedCtr,Ill.
RushUniversity
RushUniversityMedCtr-IL
RushUniversityMedCtr-IL
RushUniversityMedCtrIL
RushUniversityMedialCollege
RushUniversityMedicalCenter
RushUniversityMedicalCenter,Chicago,IL
RushUnivMedCtr
Rutgers-CommunityMedCtr,N.J.
Rutgers-JerseyCityMedicalCenter
Rutgers-NewarkBethIsraelMedicalCenter
Rutgers-NewJerseyMedicalSchool
Rutgers-NewJerseyMedicalSchool-Newark,NJ

Rutgers-NewJerseyMedicalSchool,Newark
Rutgers-NewJerseyMedicalSchool,Newark,NJ
Rutgers-R.W.JohnsonMedSchool,N.J.
Rutgers-RobertWoodJohnsonMedicalSchool-NewBrunswick,NJ
Rutgers-RobertWoodJohnsonMedicalSchool-Piscataway,NJ
Rutgers-RWJohnsonMedicalSchool-NJ
Rutgers-StBarnabasMedCtr-NJ
Rutgers–RWJohnsonMedSch,NewBrunswick,NJ
RutgersRobertWoodJohnsonMedicalSchool
SaintJosephHospital
SaintLouisUniversitySchoolofMedicine
SamaritanHealthServices
SamaritanHealthServices-Corvallis-OR
SamaritanHealthServices-Corvallis-OR
SanAntonioMilitaryMedCtr,SanAntonio,TX
SanAntonioMilitaryMedicalC
SanAntonioMilitaryMedicalCenter
SanAntonioMilitaryMedicalCenter-SanAntonio,TX
SanAntonioMilitaryMedicalCenter,SanAntonio,TX
SanAntonioMiltaryMedicalCenter
SanAntonioUniformedServicesHealthEducationConsortium(SAUSHEC)
SanMateoBehavioralHlth&RecoverySvcs-CA
SantaBarbaraCottageHosp-CA
SantaBarbaraCottageHosp-CA
SantaBarbaraCottageHospital–SantaBarbara,CA
SantaClaraValley-SanJose,CA
SantaClaraValleyMedCtr-CA
SantaClaraValleyMedCtr,Calif.(TransitionalYear,JohnPeterSmithHosp,FortWorth)
SantaClaraValleyMedicalCenter
ScheieEyeInst/UPenn
ScheieEyeInstitute,UPenn
ScheieEyeInstitute/UPenn
SchwabRehabHosp-IL
Scientist,Genentech
ScottAirForceBaseMedicalClinic
Scripps
ScrippsClinic
ScrippsClinic/GreenHospital
ScrippsClinic/GreenHospital-CA
ScrippsClinic/GreenHospital-CA

ScrippsClinic/GreenHospital,Calif.
ScrippsMercyHosp-ChulaVista-CA
ScrippsMercyHosp-SanDiego-CA
ScrippsMercyHospital-SanDiego,CA
ScrippsMercyHospital,ChulaVista,CA
SeaMarCommunityHealthCtrs-WA
SelfRegionalHealthcare-SC
SelfRegionalHealthcareSC
SidneyKimmelMedicalCollegeAtThomasJeffersonUniv
SinaiHospitalofBaltimore
SinaiHospitalofBaltimore-MD
SIU
SIUSOM&AffilHospitals,Springfield,IL
SIUSOM&AffilHosps-IL
SkagitRegionalHealth-WA
SouthBaldwinRegionalMedicalCenter
SoutheastHealth-AL
SoutheastHealth,Dothan,AL
SouthernIllinoisUniversity
SouthernIllinoisUniversitySchoolofMedicine&AffiliatedHospitals,IL
SouthernRegionalAHEC-NC
SovahHealth-Danville-VA
SpartanburgRegHealthcare-SC
SpectrumHealth/MichiganStateUniv
SpectrumHealth/MichiganStateUniversity
SpectrumHealth/MichiganStateUniversity-GrandRapids,MI
St Lukes Hosp Bethlehem PA
St.AnthonyHospitalNorth-CO
St.BarnabasMedicalCenter/Rutgers
St.Christopher'sHospitalforChildren
St.Elizabeth’sMedCtr,Mass.
St.Elizabeth'sMedicalCenter
St.Elizabeth’sMedicalCenter–Boston,MA
St.ElizabethsMedCtr-MA
St.FrancisEmoryHealthcareGA
St.Joe'sHealth-NY
St.John'sEpiscopalHospital
St.John’sRiverside
St.Joseph'sHealth
St.Joseph'sMedicalCenter

St.JosephHospital,Eureka,CA
St.JosephMercy-AnnArbor,MI
St.LouisChildren'sHospital
St.LouisChildren’sHospital
St.LouisUniversity
St.LouisUniversitySchoolofMedicine
St.LouisUnivSoM
St.Luke’sHospital
St.Luke’sHospital-Anderson–Easton,PA
St.Luke’sHospital-Anderson,PA
St.Vincent'sEast
St.Vincent’sEast,Birmingham,AL
StAgnesMedCtr-CA
StAgnesMedicalCenter-CA
StamfordHospital–ColumbiaUniv,Stamford,CT
StamfordHospital/Columbia-CT
Stanford
StanfordAdvancedResearchResidencyProgram
StanfordHealthCare
StanfordHealthCare-CA
StanfordHealthCare-CA(GlobalHealth)
StanfordHealthCare-Stanford,CA
StanfordHealthCare-CA
StanfordHealthCare–Stanford,CA
StanfordHealthCare,CA
StanfordHealthCare,Calif.
StanfordHealthCare,Calif.(Preliminary-InternalMedicine,SantaClaraValleyMedCtr,Calif.)
StanfordHealthCare,Stanford,CA
StanfordHealthCare,Stanford,CA+N1030
StanfordHealthCareCA
StanfordMedCtr
StanfordUniv
StanfordUniversity
StanfordUniversityMedicalCenter,CA
StanfordUniversityPrograms
StanfordUniversityPrograms-Stanford,Calif.
StanfordUniversityPrograms,Stanford,CA
StanfordUnivProgs-CA
StateUniversityofNewYorkDownstate
StateUniversityofNewYorkHealthScienceCenter

StateUniversityofNewYorkUpstateMedicalUniversity
StDavidsHealthcareGMETX
StElizabethMedCtr-KY
StElizabethsMedCtr-MA
StFrancisHospital-CT
StJohnHospital-MI
StJohnsEpiscopal-NY
StJohnsRiversideHospital-NY
StJohnsRiversideHospital-NY
StJosephHospitalSCLHealth-CO
StJosephHospitalSCLHealth-CO
StJosephHospitalSCLHealth,Colo.
StJosephMercy-AnnArbor-MI
StJosephsHealth-NY
StJosephsHospital-WA
StJosephsRegMedCtr-IN
StLouisChildren'sHosp,Mo.
StLouisChildrensHosp-MO
StLouisUnivSOM-MO
StLouisUnivSOM-MO
StLukesHosp-Anderson-PA
StLukesHospBethlehemPA
StLukesHospital-Anderson-Easton,PA
StLukesHospital-Bethlehem,PA
StMaryMedCtr-LongBeach-CA
StMarysMedCtr-SF-CA
StMarysMedCtrSCLHealth-CO
StonyBrookTeachHospitals-StonyBrook,NY
StonyBrookTeachHosps-NY
StonyBrookTeachingHospital-StonyBrook,NY
StonyBrookTeachingHospitals-StonyBrook,N.Y.
StrongMemorialHospitaloftheUniversityofRochesterFlaumEyeInstitute
StVincentHlthCtr-PA
StVincentsMedCtr-FL
SummaHealth/NEOMED,Rootstown
SunriseHealthGMEConsortium
SunriseHealthGMEConsortium-NV
SunriseHealthGMEConsortium,LasVegas,NV
SUNYBuffalo/EducationConsortium-Buffalo,NY
SUNYHSC-Brooklyn

SUNYHSCBrooklyn-NY
SUNYHSCBrooklyn-NY
SUNYHSCBrooklyn,N.Y.
SUNYStonyBrook
SUNYUpstateMedicalUniversity
SUNYUpstateMedUniversity
SutterHealth
SutterHealth-CA
SutterMedCtrofSantaRosa-CA
SwedishMedCtr-WA
SwedishMedCtr-WA
SwedishMedCtr/CherryHill-SeaMar,Seattle,WA
SwedishMedicalCenter
SwedishMedicalCenter-WA
TacomaFamilyMed-WA
TacomaFamilyMedicine
TacomaFamilyMedicine,Tacoma,WA
TallahasseeMemorialHealthCare
TempleUHosp,Pa.
TempleUniv/FoxChaseCancerCtr-PA
TempleUniversityHospital
TempleUniversityHospital-Philadelphia,PA
TempleUniversityHospital-Philadelphia,Pa.
TempleUniversityHospital,Philadelphia,PA
TempleUnivHosp-PA
TempleUnivHosp,Pa.
TempleUnivHospPA
TexasA&M-Bryan/CollegeStation
TexasA&M-Bryan/CollegeStation,Bryan,TX
TexasA&M-Scott&White
TexasA&MCollegeofMedicine-BaylorScottand
TexasA&MHSC/BaylorScott&White,Temple,TX
TexasA&MHSC/BaylorScottandWhite
TexasHlthResources-HarrisMethodist,FortWorth,TX
TexasInstforGradMedEduc&Research,Laredo,TX
TexasInstituteforGME&Research
TexasInstituteforGraduateMedicalEducationandResearch
TexasRehabHospital,FortWorth,TX
TexasTechUAffil-ElPaso
TexasTechUAffil-PermianBasin,Midland,TX

TexasTechUAffil-ElPaso
TexasTechUAffil,ElPaso
TexasTechUAfill,Lubbock
TexasTechUniversity,Lubbock,TX
TexasTechUniversityAffiliate,Amarillo,TX
TexasTechUniversityAffiliate,ElPaso,TX
TexasTechUniversityAffiliate,Lubbock,TX
TexasTechUniversityAffiliates-ElPaso
TexasTechUniversityHealthSciencesCenter-PermianBasin
TexomaMedicalCenter,Denison,TX
TheClevelandClinic
TheJohnsHopkinsHospital
TheJohnsHopkinsWilmerEyeInstitute
TheMayoClinicSchoolofGraduateMedicalEducation
TheOhioStateUniversity
TheUniversityofMinnesotaMedicalSchool
TheUniversityofVirginia
TheVirginiaTechCarilionClinic
ThomasHospital
ThomasJeffersonUniv
ThomasJeffersonUniv-PA
ThomasJeffersonUniv-PA/WillsEye
ThomasJeffersonUniversity
ThomasJeffersonUniversity-Philadelphia,Pa.
ThomasJeffersonUniversity–Philadelphia,PA
ThomasJeffersonUniversity,Philadelphia,PA
ThomasJeffersonUniversity/NemoursChildren’sHealth–Philadelphia,PA
ThomasJeffersonUniversityHospital
ThomasJeffersonUnivPA
TIGMER-LaredoMedicalCenter,Laredo,TX
TJU/NemoursChildrensHealthPA
TJUH
TowerHealth/DrexelUnivCOM,Pa.
TowerHealth/ReadingHospital-PA
TowerHealth/ReadingHospital-Reading,Pa.
TowerHealth/ReadingHospital-WestReading,PA
TowerHealth/St.Christopher’sHospital
TravisAFB
Tri-CountyCommissiononAlcoholandDrugAbuse
Tri-CountyCommunityonAlcoholandDrugAddiction

TridentMedicalCenter-SC
TrinityHealthAnnArborHosp-MI
TrinityHealthLivoniaHosp-MI
TriosHealth-WA
TriplerArmyBase
TriplerArmyMedicalCenter
TriplerArmyMedicalCenter,Honolulu,HI
TriplerMedicalCenter-Honolulu,HI
Tufts/NewEnglandEyeCenter
TuftsMedicalCenter
TuftsMedicalCenter-Boston,Mass.
TuftsMedicalCenter-MA
TuftsMedicalCenter-MA-Boston,MA
TuftsMedicalCenter-MA
TulaneSOM
TulaneUniversity
TulaneUniversitySchofMed,NewOrleans,LA
TulaneUniversitySchoolofMedicine
TulaneUniversitySchoolofMedicine-NewOrleans,LA
TulaneUniversitySOM
TulaneUnivSOM-LA
TulaneUnivSOM,La.
TulaneUnivSOM,NewOrleans,LA
TulaneUnivSOMLA
TulaneUSOM,La.
U Arizona COM Phoenix
U Miami/Jackson Health System-FL
U Washington Affil Hosps
U.ofArizonaCOMatTucson
UABMarnixE.HeersinkSchoolofMedicine-Huntsville
UABMedicalCenter
UAlabama
UAlabamaCCHS-Tuscaloosa
UAlabamaMedCtr-Birmingham
UAlabamaMedCtr-Montgomery
UAlabamaMedCtr-Birmingham
UAlabamaMedCtr,Birmingham
UAlabamaMedCtrBirmingham
UAlabamaMedCtrBirmingham,Birmingham,AL
UArizona

UArizonaCOM-Phoenix
UArizonaCOM-Tucson
UArizonaCOM-Tucson|UArizonaCOM-Tucson
UArizonaCOMatSouthCampus
UArizonaCOMPhoenix
UArizonaCOMTucson
UArizonaCOMTucson,Tucson,AZ
UArkansasCollofMed,LittleRock,AR
UArkansasCollofMedicine,LittleRock,AR
UArkansasCOM-LittleRock
UArkansasCOM-LittleRock
UArkansasCOM,LittleRock
UArkansasCOMLittleRock
UatBuffaloSOM-SistersofCharity,Buffalo,NY
UCaliforniaIrvine
UCaliforniaLosAngeles
UCaliforniaSanDiego
UCaliforniaSanFrancisco
UCDavis
UCDavisMedCtr
UCDavisMedCtr-CA
UCDavisMedCtr-CA
UCDavisMedicalCenter
UCDavisMedicalCenter-Sacramento,CA
UCDavisMedicalCenter–Sacramento,CA
UCDavisMedicalCenter,CA
UCDavisMedicalCenter,Sacramento,CA
UCentralFL/HCAHealthcareGME
UCFCOM/GMEConsortium-FL
UChicago
UChicagoMedCtr,Ill.
UCincinnati
UCincinnati/CincinnatiChildrensHosp
UCincinnatiMedCtr
UCincinnatiMedCtr-OH
UCincinnatiMedCtr,Cincinnati,OH
UCincinnatiMedCtr,Ohio
UCincinnatiMedCtrOH
UCincinnatiMedicalCenter
UCincinnatiWestChesterHosp-OH

UCIrnvineMedCtr
UCIrvineMedCtr
UCIrvineMedCtr-CA
UCIrvineMedCtr-CA
UCIrvineMedCtr,Calif.
UCIrvineMedCtrCA
UCIrvineMedicalCenter
UCIrvineMedicalCenter-Orange,CA
UCIrvineMedicalCenter,CA
UCLAMedCtr
UCLAMedCtr-CA
UCLAMedCtr-CA
UCLAMedCtrCA
UCLAMedicalCenter
UCLAMedicalCenter-CA
UCLAMedicalCenter-SantaMonica,CA
UCLAMedicalCenter–LosAngeles,CA
UCLAMedicalCenter,CA
UCLAMedicalCenter(Primary)
UCLASchoolofMedicine
UCLASemelInstforNeuroscience
UCLASemelInstforNeuroscience-CA
UCLASemelInstforNeuroscience,LosAngeles,CA
UCLASemelInstituteforNeuroscience
UCLASemelInstituteforNeuroscience,LosAngeles,CA
UCLASteinInstitute
UColorado
UColoradoSchofMedicine,Denver,Co
UColoradoSchoolofMedicine-Denver
UColoradoSOM
UColoradoSOM-Denver
UColoradoSOM-Denver(ChildandAdolescentPsychiatry)
UColoradoSOM-Denver
UColoradoSOM,Denver
UColoradoSOMDenver,Aurora,CO
UConnecticutSchoolofMedicine
UConnecticutSchoolofMedicine,Farmington
UCRiversideSOM-CA
UCSanDiego
UCSanDiegoMedCtr

UCSanDiegoMedCtr-CA
UCSanDiegoMedCtr-CA
UCSanDiegoMedCtr,Calif.
UCSanDiegoMedicalCenter
UCSanDiegoMedicalCenter-SanDiego,CA
UCSanDiegoMedicalCenter,CA
UCSanDiegoMedicalCenter(Research)
UCSanFrancisco
UCSanFrancisco-CA
UCSanFrancisco-EastBay
UCSanFrancisco-EastBay-CA
UCSanFrancisco-EastBay,CA
UCSanFrancisco-Fresno-CA
UCSanFrancisco-SanFrancisco,CA
UCSanFrancisco-CA
UCSanFrancisco,CA
UCSanFrancisco,Calif.
UCSanFrancisco,SanFrancisco,CA
UCSanFrancisco(TransitionalYear,SantaClaraValleyMedCtr,Calif.)
UCSanFranciscoMedicalCenter
UCSD
UCSDMedCtr
UCSF
UCSF-SanFrancisco,CA
UCSF–SanFrancisco,CA
UCSFBenioffChildren'sHospitalOakland
UCSFMedCtr
UFlorida
UFlorida-Gainesville
UFloridaCOM-ShandsHosp
UFloridaCOM-ShandsHosp/Gainesville
UFloridaCOM-ShandsHospital,Gainesville,FL
UFloridaCOM-Jacksonville
UFloridaCOM-ShandsHosp
UFloridaCOM–ShandsHosp,Gainesville,FL
UFloridaCOM–ShandsHospital,Gainesville,FL
UFloridaCOM,Jacksonville
UFloridaCOMJacksonville
UFloridaCOMJacksonville,Jacksonville,FL
UFloridaCOMShandsHosp

UFloridaCOMShandsHosp,Gainesville,FL
UHosps,Jackson,Miss.
UHS-WilsonMedCtr-NY
UHSSCalif.MedEdConsortium
UHSSoCaliforniaMedEdConsortium
UIC-IllinoisEyeandEarInfirmary
UIC/MetroGroupHospitals-IL
UIllinoisCollegeofMedicine-Chicago
UIllinoisCOM
UIllinoisCOM-Chicago
UIllinoisCOM-PeoriaOSF
UIllinoisCOM-Chicago
UIllinoisCOM-PeoriaOSF
UIllinoisCOM,Chicago
UIllinoisCOMChicago
UIowaHospitalsandClinics,IowaCity,IA
UIowaHospsandClinics
UIowaHospsandClinics,IowaCity,IA
UKansasSOM
UKansasSOM-KansasCity
UKansasSOM-Wichita
UKansasSOM,KansasCity
UKansasSOMKansasCity
UKansasSOMKansasCity,KansasCity
UKentuckyMedCtr
UKentuckyMedCtr,Lexington
UKentuckyMedCtr,Lexington,KY
UKentuckyMedicalCenter,Lexington,KY
ULouisvilleSOM-KY
ULouisvilleSOM-Ky.
UMarylandMercyMedCtr
UMarylandPrinceGeorgesHospCtr
UMassachusettsMedSchool
UMassChan-Baystate-MA
UMassChan-Baystate-MA
UMassChanMedicalSchool
UMassChanMedicalSchool-MA
UMassChanMedicalSchool-MA
UMassChanMedicalSchool,Worcester,MA
UMassChanMedSchool,Mass.

UMDNJ-NewJerseyMed-Newark
UMiami/BascomPalmer
UMiami/BascomPalmer(Preliminary-InternalMedicine,UMiami/JacksonHealthSystem,Fla.)
UMiami/JacksonHealthSystem-FL
UMiami/JacksonHealthSystem-FL
UMiami/JacksonHealthSystem,Fla.
UMiami/JacksonHealthSystem,Fla.(TransitionalYear,ProvidenceSacredHeartMedCtr,Wash.)
UMiami/JacksonHealthSystem,Miami,FL
UMiamiMSOM
UMiamiMSOM/HolyCross-FL
UMiamiMSOM/PalmBeachRegCampus-FL
UMichigan
UMichiganHospital-AnnArbor
UMichiganHospitals-AnnArbor
UMichiganHospitals,AnnArbor,MI
UMichiganHosps
UMichiganHosps-AnnArbor
UMichiganHosps-AnnArbor(Chelsea)
UMichiganHosps-AnnArbor(Ypsilanti)
UMichiganHosps-AnnArbor
UMichiganHosps,AnnArbor
UMichiganHosps,AnnArbor(TransitionalYear,UTAustinDellMedSchool)
UMichiganHospsAnnArbor
UMinnesota
UMinnesota-Minneapolis
UMinnesotaMedicalSchool
UMinnesotaMedSchool
UMissouri-KansasCity
UMissouriKansasCity
UMKC
UNC
UNevadaAffilHosps-LasVegas
UNevadaLasVegasSOM
UNevadaRenoSOM
UNewMexicoSOM
Uni.OfTennessee
UnityHealth-AR
UnityHealth,Searcy,AR
Univ of Texas Health Sci Ctr, San Antonio, TX
Univ of Texas Medical Branch, Galveston, TX

Univ of Texas Medical School, Houston, TX
Univ.ofConnecticutHealthCenter
Univ.ofKansasSchofMedicine,KansasCity,KS
Univ.ofPittsburghMedCtr
UnivCalifornia-Irvine
UniveristyofSouthernCalifornia
UniversityatBuffaloSchoolofMedicine
UniversityatBuffaloSchoolofMedicine-Buffalo,NY
UniversityatBuffaloSchoolofMedicine–Buffalo,NY
UniversityatBuffaloSOM-NY
UniversityatBuffaloSOM-NY
UniversityatBuffaloSOM,Buffalo,NY
UniversityHospitals,Jackson,MS
UniversityHosps-Columbia-MO
UniversityHosps-Jackson-MS
UniversityHospsJacksonMS
UniversityMinnesotaMedSchool
UniversityNewMexicoSchoolofMedicine
UniversityNorthCarolinaHospitals
UniversityofAlabama
UniversityofAlabama-Birmingham
UniversityofAlabamaatBirminghamMarnixE.HeersinkSchoolofMedicine-Huntsville
UniversityofAlabamaBirmingham-Birmingham,AL
UniversityofAlabamaCollegeofCommunityHealthSciences
UniversityofAlabamaMedicalCenter(Birmingham)
UniversityofAlabamaSchoolofMedicine-Huntsville
UniversityofArizonaCollegeofMedicine-Phoenix
UniversityofArizonaCollegeofMedicine-Phoenix,AZ
UniversityofArizonaCollegeofMedicine-Tucson,AZ
UniversityofArizonaCollegeofMedicineSouthCampus
UniversityofArizonaCollofMed,Tucson,AZ
UniversityofArkansas
UniversityofArkansasCollegeofMedicine-Little
UniversityofArkansasforMedicalSciencesCollegeof
UniversityofArkansasforMedicalSciencesCollegeofMedicine
UniversityofCaliforna-SanFrancisco
UniversityofCalifornia-DavisMedicalCenter-Sacramento,CA
UniversityofCalifornia-LosAngeles
UniversityofCalifornia-LosAngelesMedicalCenter-LosAngeles,CA
UniversityofCalifornia-SanDiego

UniversityofCalifornia-SanDiegoMedicalCenter-SanDiego,CA
UniversityofCalifornia-SanFrancisco
UniversityofCalifornia–Irvine,Orange,CA
UniversityofCalifornia,Irvine
UniversityofCalifornia,SanDiego
UniversityofCalifornia,SanDiego(UCSD,PSTP)
UniversityofCalifornia,SanFrancisco
UniversityofCalifornia,SanFrancisco(UCSF)
UniversityofCalifornia;SanDiego
UniversityofCaliforniaDavis
UniversityofCaliforniaDavisMedicalCenter
UniversityofCaliforniaIrvineMedicalCenter
UniversityofCaliforniaLosAngeles
UniversityofCaliforniaLosAngelesMedicalCenter
UniversityofCaliforniaLosAngelesMedicalCenter-LosAngeles,Calif.
UniversityofCaliforniaSanDiegoMedCenter-SanDiego,CA
UniversityofCaliforniaSanDiegoMedicalCenter
UniversityofCaliforniaSanFrancisco
UniversityofCaliforniaSanFrancisco-EastBay-Oakland,CA
UniversityofCaliforniaSanFrancisco-SanFrancisco,CA
UniversityofCaliforniaSanFranciscoMedicalCenter
UniversityofCentralFlorida
UniversityofCentralFloridaCollegeofMedicine/GraduateMedicalEducationConsortium
UniversityofChicago
UniversityofChicagoMedCenter,Chicago,IL
UniversityofChicagoMedCtr,Chicago,IL
UniversityofChicagoMedicalCenter
UniversityofChicagoMedicalCenter-Chicago,IL
UniversityofChicagoMedicalCenter–Chicago,IL
UniversityofChicagoMedicalCenter,IL
UniversityofChicagoMedicine
UniversityofCincinnati
UniversityofCincinnatiMedCtr,Cincinnati,OH
UniversityofCincinnatiMedicalCenter
UniversityofCincinnatiMedicalCenter-Cincinnati,OH
UniversityofCincinnatiMedicalCenter,OH
UniversityofColorado
UniversityofColorado–AnschutzMedicalCampus–Aurora,CO
UniversityofColorado–Denver
UniversityofColoradoAnschutzMedicalCampus

UniversityofColoradoSchofMed,Aurora,CO
UniversityofColoradoSchofMed,Denver,CO
UniversityofColoradoSchoolofMedicine
UniversityofColoradoSchoolofMedicine-Aurora,CO
UniversityofColoradoSchoolofMedicine-Denver
UniversityofColoradoSchoolofMedicine-Denver,CO
UniversityofColoradoSchoolofMedicine-Denver
UniversityofColoradoSchoolofMedicine–Aurora,CO
UniversityofColoradoSchoolofMedicine,Denver
UniversityofColoradoSOM-Denver
UniversityofConnecticutHealthCenter–Hartford,CT
UniversityofConnecticutSchoolofMedicine
UniversityofConnecticutSchoolofMedicine-Farmington,CT
UniversityofConnecticutSchoolofMedicine-Hartford,CT
UniversityofFlorida
UniversityofFlorida-Shands
UniversityofFloridaCollegeofMedicine
UniversityofFloridaCollegeofMedicine-Jacksonville
UniversityofFloridaCollegeofMedicine-Shands
UniversityofFloridaCollegeofMedicine-ShandsHospital
UniversityofFloridaCollegeofMedicine-ShandsHospital-Gainesville,FL
UniversityofFloridaCollegeofMedicine-ShandsHospital
UniversityofFloridaHealthScienceCenterJacksonville
UniversityofFloridaHealthShandsHospital
UniversityofHawaii
UniversityofHawaii-Honolulu,HI
UniversityofHawaii(PrimaryCare)
UniversityofIllinoisCollegeofMedicine
UniversityofIllinoisCollegeofMedicine-Chicago,IL
UniversityofIllinoisCollegeofMedicine,Chicago
UniversityofIllinoisCOM
UniversityofIowaCarverCollegeofMedicine
UniversityofIowaHospitals-IowaCity,IA
UniversityofIowaHospitalsandClinics
UniversityofIowaHospitalsandClinics-IowaCity,IA
UniversityofIowaHospitalsandClinics,IowaCity,Iowa
UniversityofKansasMedicalCenter
UniversityofKansasSchoolofMedicine
UniversityofKansasSchoolofMedicine-Wichita
UniversityofKentucky

UniversityofKentuckyCollegeofMedicine
UniversityofKentuckyMedicalCenter
UniversityofKentuckyMedicalCenter-Lexington,KY
UniversityofLouisville
UniversityofLouisvilleSchoolofMedicine
UniversityofMaryland
UniversityofMarylandMedCtr
UniversityofMarylandMedicalCenter
UniversityofMarylandMedicalCenter-Baltimore,MD
UniversityofMarylandMedicalCenter-Baltimore,Md.
UniversityofMassachusetts-Chan-Baystate,Springfield,MA
UniversityofMassachusettsChanMedicalSchool
UniversityofMassachusettsChanMedicalSchool,Worcester,MA
UniversityofMassachusettsMedicalSchool
UniversityofMassachusettsMedicalSchool-Worcester,Mass.
UniversityofMiami
UniversityofMiami,BascomPalmer
UniversityofMiami/BascomPalmerEyeInstitute
UniversityofMiami/JacksonHealthSystem
UniversityofMiamiHealthSystem/JacksonHealth
UniversityofMichigan
UniversityofMichigan-AnnArbor
UniversityofMichiganHospital
UniversityofMichiganHospital-AnnArbor,MI
UniversityofMichiganHospitalatAnnArbor
UniversityofMichiganHospitals
UniversityofMichiganHospitals-AnnArbor
UniversityofMichiganHospitals-AnnArbor-AnnArbor,Mich.
UniversityofMichiganHospitals-AnnArbor,MI
UniversityofMichiganHospitals,AnnArbor
UniversityofMichiganHospitals,AnnArbor,MI
UniversityofMichiganMedicalCenter
UniversityofMinnesota
UniversityofMinnesotaMedicalCenter
UniversityofMinnesotaMedicalSchool
UniversityofMinnesotaMedicalSchool-Minneapolis,MN
UniversityofMississippiMedicalCenter
UniversityofMissouri
UniversityofMissouri-KCPrograms
UniversityofMontana

UniversityofMontana,Missoula,MT
UniversityofNebraska
UniversityofNebraskaMedCtr
UniversityofNebraskaMedicalCenter
UniversityofNebraskaMedicalCenter-Omaha,NE
UniversityofNevadaRenoSchoolofMedicine-Reno,NV
UniversityofNewMexico
UniversityofNewMexicoSchoolofMedicine
UniversityofNorthCarolina
UniversityofNorthCarolinaHospitals
UniversityofNorthCarolinaHospitals-ChapelHill,N.C.
UniversityofNorthCarolinaHospitals-ChapelHill,NC
UniversityofNorthCarolinaHospitals–ChapelHill,NC
UniversityofNorthCarolinaSchoolofMedicine
UniversityofOklahomaCollegeofMedicine
UniversityofOklahomaCollegeofMedicine-Tulsa
UniversityofOttawa,Canada
UniversityofPennsylvania
UniversityofPennsylvania/Children'sHospitalofPhiladelphia
UniversityofPennsylvania/Pennsy
UniversityofPennsylvaniaMedicalCenterMedicalEducation,PA
UniversityofPittsburgh
UniversityofPittsburgh-Hamot
UniversityofPittsburghMedicalCenter
UniversityofPittsburghMedicalCenter-AltoonaHospital-Altoona,Pa.
UniversityofPittsburghMedicalCenter-MedicalEducation-Pittsburgh,Pa.
UniversityofPittsburghMedicalCenter-MercyHospital-Pittsburgh,Pa.
UniversityofPittsburghMedicalCenter-Pittsburgh,PA
UniversityofPittsburghMedicalCenter(UPMC)
UniversityofPittsburgMedicalCenter(UPMC)Medical
UniversityofPittsburgMedicalCenter(UPMC)MedicalEducation
UniversityofRochester
UniversityofRochester/StrongMemorial
UniversityofRochester/StrongMemorial-Rochester,N.Y.
UniversityofRochester/StrongMemorial-Rochester,NY
UniversityofRochester/StrongMemorial,NY
UniversityofRochester/StrongMemorialHospital
UniversityofRochester/StrongMemorialHospital-Rochester,NY
UniversityofRochesterFlaumEyeInstitute
UniversityofRochesterMedicalCenter-Rochester,NY

UniversityofSanDiegoMedicalCenter
UniversityofSouthAlabama
UniversityofSouthAlabama,Mobile,AL
UniversityofSouthAlabamaHospitals
UniversityofSouthCarolina/PrismaHealth
UniversityofSouthernCalifornia
UniversityofSouthernCalifornia-LosAngeles,CA
UniversityofSouthernCalifornia,LosAngeles,CA
UniversityofSouthernCaliforniaRoskiEyeInstitute
UniversityofSouthFlorida
UniversityofSouthFloridaMorsaniCollegeofMedicine
UniversityofSouthFloridaMorsaniCollegeofMedicine-Tampa,FL
UniversityofSouthFloridaMorsaniCollegeofMedicineatTampa-Tampa,Fla.
UniversityofTennessee
UniversityofTennessee/AscensionSt.ThomasHealth
UniversityofTennesseeCollegeofMedicineChattanooga
UniversityofTennesseeGraduateSchoolofMedicine
UniversityofTennesseeHealthScienceCenter
UniversityofTexas
UniversityofTexas-Southwestern
UniversityofTexas,Southwestern
UniversityofTexasatAustin/DellMedicalSchool-Austin,TX
UniversityofTexasatAustinDellMedicalSchool
UniversityofTexasatAustinDellMedicalSchool-Austin,Texas
UniversityofTexasHealthScienceCenter
UniversityofTexasHealthScienceCenteratHouston
UniversityofTexasHealthScienceCenteratSanAntonio
UniversityofTexasHouston
UniversityofTexasMDAndersonCancerCenter
UniversityofTexasMedicalBranch,Galveston
UniversityofTexasMedicalBranch,Galveston,TX
UniversityofTexasMedicalBranchHospitals
UniversityofTexasMedicalSchool-Houston
UniversityofTexasMedicalSchool-Houston,TX
UniversityofTexasMedicalSchool,Houston
UniversityofTexasMedicalSchool,Houston,TX
UniversityofTexasSouthwestern
UniversityofTexasSouthwesternMedical
UniversityofTexasSouthwesternMedicalCenter
UniversityofTexasSouthwesternMedicalSchool

UniversityofTexasSouthwesternMedicalSchool-Dallas,TX
UniversityofTexasSouthwesternMedicalSchool,Dallas
UniversityofToledo
UniversityofToledo-OH
UniversityofToledoMedicalCenter
UniversityofUtah
UniversityofUtahHealth
UniversityofUtahHealth-SaltLakeCity,UT
UniversityofUtahHealth-SaltLakeCity,Utah
UniversityofUtahHealth–SaltLakeCity,UT
UniversityofUtahHealth,SaltLakeCity,UT
UniversityofVermont
UniversityofVermontMedicalCenter
UniversityofVermontMedicalCenter-Burlington,VT
UniversityofVermontMedicalCenter-Burlington,Vt.
UniversityofVirginia
UniversityofVirginia-Charlottesville,VA
UniversityofVirginia-Charlottesville,Va.
UniversityofVirginia–Charlottesville,VA
UniversityofWashington
UniversityofWashingtonAffiliatedHospitals
UniversityofWashingtonAffiliatedHospitals-Seattle,WA
UniversityofWashingtonAffiliatedHospitals,Seattle,WA
UniversityofWashingtonAffiliatedHospitals(Primary)
UniversityofWashingtonAffiliatedHospitals(Research)
UniversityofWashingtonAffiliatedHospitals(ResearchTrack)
UniversityofWashingtonAffiliateHospitals
UniversityofWashingtonAffiliateHospitals-Seattle,Wash.
UniversityofWashingtonHospital
UniversityofWashingtonMedicalCenter
UniversityofWisconsin
UniversityofWisconsin,Madison
UniversityofWisconsinHospital
UniversityofWisconsinHospitalandClinics
UniversityofWisconsinHospitalandClinics–Madison,WI
UniversityofWisconsinHospitalsandClinics
UniversityofWisconsinSchoolofMedicineandPublicHealth
UniversityTennesseeHealthScienceCenter
UnivHospsCommunityConsortium-OH
UnivKansasMedCtr

UnivNorthCarolinaHospitals,ChapelHill,NC
UnivofAlabamaMedCtr,Birmingham,AL
UnivofArizonaCollegeofMedicine,Tucson,AZ
UnivofArkansasCollofMed,LittleRock,AR
UnivofArkansasCOM,LittleRock,AR
UnivofCalifornia-SanDiego
UnivofCalifornia,SanFrancisco,CA
UnivofCentralFloridaCollegeofMed,Ocala,FL
UnivofCentralFloridaCollofMed,Ocala,FL
UnivofChicago
UnivofChicagoMedCtr
UnivofChicagoMedCtr-IL
UnivofChicagoMedCtr-IL
UnivofChicagoMedCtrIL
UnivofCincinnatiMedCtr
UnivofColorado
UnivofColoradoMedCtr
UnivofColoradoSoM
UnivofFloridaCoM
UnivofIowaHospitals&Clinics,IowaCity,IA
UnivofIowaHospsandClinics
UnivofKansasSchofMed,KansasCity,KS
UnivofKansasSoM
UnivofKansasSOM,KansasCity,KS
UnivofLouisvilleSchofMed,Louisville,KY
UnivofMaryland
UnivofMassChanMedSch
UnivofMichigan
UnivofMichiganHospitals
UnivofMinnesota
UnivofMinnesotaMedSchool
UnivofMissouri-KCPrograms
UnivofMissouri–KansasCity,MO
UnivofMissouri,KansasCity,MO
UnivofMO-KCPrograms
UnivofNebraskaMedicalCenter,Omaha,NE
UnivofNewMexicoSchofMed,Albuquerque,NM
UnivofNewMexicoSOM,Albuquerque,NM
UnivofNorthCarolinaHospitals
UnivofNorthCarolinaHospitals,ChapelHill,NC

UnivofOklahomaCollofMed,OklahomaCity,OK
UnivofPittsburghMedCtr
UnivofRochester/StrongMemorial,Rochester,NY
UnivofTexasatAustinDellMedSch,Austin,TX
UnivofTexasHealthSciCtr,SanAntonio,TX
UnivofTexasHealthSciCtr,Tyler,TX
UnivofTexasHealthScienceCenter,Tyler,TX
UnivofTexasHlthSciCtr,SanAntonio,TX
UnivofTexasMedicalBranch,Galveston,TX
UnivofTexasMedicalSch-HH/LBJ,Houston,TX
UnivofTexasMedicalSchool,Houston,TX
UnivofTexasRioGrandeValley,Harlingen,TX
UnivofTexasSouthwesternMedSch,Dallas,TX
UnivofToronto
UnivofUtahHealth
UnivofUtahHealth,SaltLakeCity,UT
UnivofVermontMedicalCenter
UnivofVirginiaMedCtr
UnivofWashington
UnivofWisconsin,Madison
UnivofWyoming-Casper
UnivofWyomingCheyenne
UnivPittsburghMedCtr
UnivTexasSouthwesternMedSch
UnivWashingtonAffilHosps
UNLV/NellisAFB
UNorthCarolina
UNorthCarolinaHospitals
UNorthCarolinaHospitals,ChapelHill,NC
UNorthCarolinaHosps
UNorthDakotaSOM
UofArizonaCollegeofMedicine,Tucson,AZ
UofArkansasCollofMed,LittleRock,AR
UofCalifornia-LosAngeles
UofCentralFloridaCollMed,Ocala,FL
UofHawaii
UofMissouri-KC/CBM
UofNorthCarolina-ChapelHill
UofOklahomaCollofMed,OklahomaCity,OK
UofOklahomaCollofMedicine,Tulsa,OK

UofTexasatAustinDellMedicalSch,Austin,TX
UofTexasHealthSciCtr,SanAntonio,TX
UofTexasHealthScienceCtr-Tyler,Athens,TX
UofTexasMedicalBranch-Methodist,Galveston,TX
UofTexasRioGrandeValley/VBMC,Harlingen,TX
UofUtahHealth
UOklahomaCollofMed,OklahomaCity,OK
UOklahomaCOM
UOklahomaCOM-OKCity
UOklahomaCOM,OklahomaCity
UOklahomaCOM,Tulsa
UOklahomaMedicalCenter
Upenn
UPenn/ChildrensHospofPhiladelphia
UPennsylvania
UPittsburgh
UPMC
UPMC Medical Education
UPMC(Pittsburgh)
UPMCHamotMedicalCenter
UPMCHarrisburg-Harrisburg,PA
UPMCMcKeesport
UPMCMedicalEducation
UPMCMedicalEducation-PA
UPMCMedicalEducation-Pittsburgh,PA
UPMCMedicalEducation–Pittsburgh,PA
UPMCMedicalEducation,Pa.
UPMCMedicalEducation,Pittsburgh
UPMCMedicalEducation(General)
UPMCMedicalEducation(Research)
UPMCMedicalEducationPA
UPMCMercyHospital
UPMCMercyHospital-PA
UPMCSt.Margaret
UPMCStMargaret
UPMCStMargaret-PA
UPMCStMargaret-Pittsburgh,PA
URochester
URochester/StrongMem-NY
URochester/StrongMemorial

URochester/StrongMemorial-NY
URochester/StrongMemorial-NY
URochester/StrongMemorial,N.Y.
URochester/StrongMemorial,Rochester,NY
URochester/StrongMemorialHosp,Rochester,NY
USC
USCRoskiEyeInstitute
USouthAlabamaHospitals
USouthAlabamaHospitals,Mobile,AL
USouthernCalifornia
USouthernCalifornia(Preliminary-InternalMedicine,BaylorUMedCtr,Dallas)
USouthernCalifornia/KeckMedicine
USouthFloridaCOM-Tampa
USouthFloridaMorsaniCOM-Tampa
USouthFloridaMorsaniCOM,Tampa,FL
USouthFloridaMorsaniCOMTampa
UtahHealthCareInstitute/St.Mark's
UtahValleyRegMedCtr
UTAscensionSt.Thomas,Murfreesboro,TN
UTAscensionStThomas-TN
UTAscensionStThomasTN
UTAustinDellMedSchool
UTAustinDellMedSchool(Preliminary-InternalMedicine,VirginiaMasonMedCtr,Wash.)
UTAustinDellMedSchool(TransitionalYear,VirginiaMasonMedCtr,Wash.)
UTenn.GradSOM,Knoxville
UTenn.HealthSciCtr,Memphis
UTennessee
UTennesseeCOM
UTennesseeCOM-Chattanooga
UTennesseeCOMChattanooga
UTennesseeCOMChattanooga,Chattanooga,TN
UTennesseeGradSOM-Knoxville
UTennesseeGradSOM,Knoxville,TN
UTennesseeGradSOMKnoxville
UTennesseeGradSOMKnoxville,Knoxville,TN
UTennesseeHealthSciCtr-Memphis
UTennesseeHealthSciCtr-Memphis
UTennesseeHealthSciCtr,Memphis
UTennesseeHealthSciCtr,Memphis,TN
UTennesseeHealthSciCtrMemphis

UTennesseeHealthScienceCenter-Memphis
UTennesseeHealthScienceCtr,Memphis,TN
UTexas
UTexasatAustinDellMedicalSchool
UTexasatAustinDellMedicalSchool,Austin,TX
UTexasatAustinDellMedSch-Ascension,Austin,TX
UTexasHealthScienceCenter,Tyler,TX
UTexasHealthScienceCtr-Tyler,Pittsburg,TX
UTexasHlthSciCtr–VADoD,Houston,TX
UTexasHSC-SanAntonio
UTexasHSC-Tyler
UTexasHSCSanAntonio
UTexasHSCSanAntonio,SanAntonio,TX
UTexasMDAndersonCancerCtr
UTexasMedBranch-Galveston
UTexasMedicalSchool-Houston
UTexasMedicalSchool,Houston,TX
UTexasMedSch-Houston
UTexasMedSch-Houston,TX
UTexasMedSch-Houston
UTexasMedSch–Houston,TX
UTexasMedSchHouston
UTexasMedSchHouston,Houston,TX
UTexasMedSchool,Houston,TX
UTexasRioGrandeValley/DHR,Edinburg,TX
UTexasSouthwesternMedicalSchool
UTexasSouthwesternMedicalSchool-Dallas
UTexasSouthwesternMedSch
UTexasSouthwesternMedSch-Dallas
UTexasSouthwesternMedSch-Dallas
UTexasSouthwesternMedSch,Dallas,TX
UTexasSouthwesternMedSchDallas
UTHouston
UTHSC-SanAntonio
UTHSC,SanAntonio
UTHSC,Tyler
UTMDAndersonCancerCtr
UTMedBranch-Galveston
UTMedBranch,Galveston
UTMedSchool,Houston

UTRioGrandeValley
UTSanAntonio
UTSouthwestern
UTSouthwestern-Dallas
UTSouthwestern(Preliminary-InternalMedicine,BaylorUMedCtr,Dallas)
UTSouthwestern(Preliminary-InternalMedicine,PresbyterianHosp,Dallas)
UTSouthwestern(Preliminary-InternalMedicine,UTSouthwestern)
UTSouthwestern(TransitionalYear,BaylorScott&WhiteAllSaintsMedCtr,Texas)
UTSouthwestern(TransitionalYear,HCAHoustonHealthcare/UHouston)
UTSouthwestern(TransitionalYear,JohnPeterSmithHosp,FortWorth)
UTSouthwestern(Transitionalyear,UTAustinDellMedSchool)
UTSouthwesternMedicalSchool
UUtah
UUtahAffilHospitals
UUtahHealth
UUtahHealth(Preliminary-InternalMedicine,AmitaSt.JosephHosp,Ill.)
UUtahHealth(TransitionalYear,IntermountainMedCtr,Utah)
UVirginia
UWashAffilHosps
UWashAffilHosps(Preliminary-InternalMedicine,UWashAffilHosps)
UWashAfillHosps
UWashington
UWashingtonAffilHospitals,Seattle,WA
UWashingtonAffilHosps
UWashingtonAffiliatedHospitals
UWashingtonAffiliatedHospitals,Seattle,WA
UWashingtonBoise-ID
UWiscHospandClinics
UWisconsinClinicalScienceCtr
UWisconsinHospitalandClinics
UWisconsinSOM&PublicHealth,Madison,WI
UWisconsinSOMandPublicHealth
UWisconsinSOMandPublicHealth,Madison,WI
VAGreaterLAHealthSystem
VAGreaterLAHealthSystems
VAGreaterLAHlthSys-CA
ValleyHealthSystem-NV
ValleyHealthSystem-NV
ValleyHospMedCtr-NV
ValleyMedCtr-WA

ValleyMedCtr,Wash.
ValleyMedicalCenter
Vanderbilt&FacebookRealityLabs
VanderbiltUMedCtr,T1603:T1604Tenn.
VanderbiltUMedCtr,Tenn.
VanderbiltUniv
VanderbiltUniversity
VanderbiltUniversityMedicalCenter
VanderbiltUniversityMedicalCenter-Nashville,TN
VanderbiltUniversityMedicalCenter–Nashville,TN
VanderbiltUniversityMedicalCenter,Nashville,TN
VanderbiltUniversityMedicalCenter,TN
VanderbiltUniversityMedicalCtr,Nashville,TN
VanderbiltUniversitySchoolofMed.
VanderbiltUniversitySchoolofMedicine
VanderbiltUnivMedCtr-TN
VanderbiltUnivMedCtr-TN
VanderbiltUnivMedCtr,TN
VanderbiltUnivMedCtrTN
VCU-ShenandoahValley-VA
VCUFamilyPractice,FrontRoyal,VA
VCUHealthSystem
VenturaCountyMedCtr-CA
VenturaCountyMedCtr-CA
VenturaCountyMedCtr,Calif.
VenturaCountyMedicalCenter
VenturaCountyMedicalCenter,Ventura,CA
VidantMedCtr/EastCarolinaUniv
VidantMedCtr/EastCarolinaUniv-NC
VidantMedCtr/EastCarolinaUnivNC
VirginiaCommonwealthUHlthSys
VirginiaCommonwealthUHlthSys|VirginiaCommonwealthUHlthSys
VirginiaCommonwealthUHlthSystem
VirginiaCommonwealthUni
VirginiaCommonwealthUniversity
VirginiaCommonwealthUniversityHealthSystem
VirginiaCommonwealthUniversityHealthSystem-Richmond,VA
VirginiaCommonwealthUniversityHealthSystem-Richmond,Va.
VirginiaCommonwealthUniversityHealthSystems
VirginiaCommonwealthUnivHealthSystem,Richmond,VA

VirginiaMasonFranciscanHealth-Seattle,WA
VirginiaMasonFranciscanHealth-WA
VirginiaMasonMedCtr-WA
VirginiaMasonMedicalCenter
VirginiaMasonMedicalCenter,WA
VirginiaTech
VirginiaTechCarilionSchofMed,Roanoke,VA
WakeForestBaptistMedCtr
WakeForestBaptistMedCtr-NC
WakeForestBaptistMedCtr-NC
WakeForestBaptistMedCtr,Winston-Salem,NC
WakeForestBaptistMedCtrNC
WakeForestBaptistMedicalCenter
WakeForestBaptistMedicalCenter-Winston-Salem,N.C.
WakeForestBaptistMedicalCenter-Winston-Salem,NC
WakeForestBaptistMedicalCenter-Winston-Salmen,NC
WakeForestBaptistMedicalCenter,Winston-Salem,NC
WakeForestMedicalCenter
WakeForestUniversity
WakeForestUniversityBaptistMedicalCenter
WakeForestUniversitySchoolofMedicine
WakeForestUnivSch.ofMed.
WakeMedHealthandHospitals-NC
WalterReed
WALTERREEDARMYMEDCNTRWASH
WalterReedArmyMedicalCenter-Bethesda,Md.
WalterReedMedicalCenter
WalterReedMilitaryMedicalCenter
WalterReedNat’lMilitaryMedCtr,Bethesda,MD
WalterReedNationalMilitaryMedCtr
WalterReedNationalMilitaryMedicalCenter
WalterReedNationalMilitaryMedicalCenter-Bethesda,MD
WalterReedNatlMilitaryMedCtr,Bethesda,MD
WalterReedNtlMedctr
WashingtonHospital
WashingtonStateUniv
WashingtonUniv
WashingtonUniversity
WashingtonUniversity-BarnesJewish
WashingtonUniversity/Barnes-JewishHospital

WashingtonUniversityatSt.Louis
WashingtonUniversityinSt.Louis
WashingtonUniversityMedicalCenter
WashingtonUniversityMedicalCenter/BarnesJewishHospital
WashingtonUniversityofSt.LouisChildren'sHospital
WashU
WashU/Barnes-Jewish
WashU/Barnes-Jewish-MO
WashU/St.LouisChildren’sHospital
WayneStateUniv/HenryFordHospital
WayneStateUniversity
WayneStateUniversitySOM-MI
WellspanHealthGoodSamaritanHospital-Lebanon,PA
WellspanHealthYorkHosp-PA
WellspanHealthYorkHospital
WellStarKennestoneRegionalMedicalCenter
WellStarKennestoneRegionalMedicalCenter-Marietta,GA
WellStarKennestoneRegMedCtr-GA
WellStarKennestoneRegMedCtrGA
WestchesterMedicalCtr-NY
WesternMichiganUniversityStrykerSchoolofMedicine
WesternMichiganUnivStrykerSOM
WesternPennsylvaniaHosp
WesternPennsylvaniaHospital
WesternReserveHealthEd/NEOMED-OH
WestVirginiaEyeInstitute
WestVirginiaUniversity
WestVirginiaUniversityEyeInstitute
WestVirginiaUniversitySchoolofMedicine
WestVirginiaUniversitySchoolofMedicine-Morgantown,W.Va.
WestVirginiaUniversitySOM
WestVirginiaUniversitySOM,Morgantown,WV
WestVirginiaUnivSoM
WestVirginiaUSOM
White Medical Center
WhiteMedicalCenter
WILLIAMBEAUMONTARMYHOSP
WilliamBeaumontArmyMedicalCenter-ElPaso,TX
WillsEyeHospital
WillsEyeHospital-ThomasJefferson

Wilmer-JohnsHopkins
Wilmer–JohnsHopkinsUniversity,Baltimore,MD
WiNCGMEConsortium-WI
WorkingTitleSimulations
Wright-PattersonAFB
Wright-PattersonAirForceBase
WrightPattersonAFB
WrightPattersonAirForceBase
WrightPattersonAirForceBase-WrightPattersonAFB,Ohio
WrightPattersonMedicalCenter
WrightState
WrightStateU-WrightStateAirForceBase
WrightStateUnivBoonshoftSOM-OH
WrightStateUniversityBoonshoftSchoolofMedicine
WrightStateUniversityBoonshoftSOM
WSU/DetroitMedCtr-MI
Yale
Yale-NewHavenHosp-CT
Yale-NewHavenHosp-CT(PSTP)
Yale-NewHavenHosp,Conn.
Yale-NewHavenHosp,Conn.(Preliminary-InternalMedicine,UTHSC,SanAntonio)
Yale-NewHavenHosp,Conn.(TransitionalYear,BrookwoodBaptistHealth,Ala.)
Yale-NewHavenHospital
Yale-NewHavenHospital-CT
Yale-NewHavenHospital-NewHaven,Conn.
Yale-NewHavenHospital-NewHaven,CT
Yale-NewHavenHospital,CT
Yale-NewHavenHospital,NewHaven
Yale-NewHavenHospital,NewHaven,CT
Yale-NewHavenHospital(Primary)
Yale-NewHavenHospital
YaleNewHavenHealth
YaleNewHavenHosp
YaleNewHavenHospCT
YaleNewHavenHospital
YaleUniv
YaleUniversity
ZucherSchoolofMedicineatHofstra/Northwell
ZuckerSchoolofMedicine-Northwell
ZuckerSchoolofMedicine-NorthwellCohenChildren's-NewHydePark,N.Y.

ZuckerSchoolofMedicine-NorthwellCohenChildrensHospital-NewHydePark,NY
ZuckerSchoolofMedicine-NorthwellNorthShore/LongIslandJewishMedicalCenter-GreatNeck,N.Y.
ZuckerSchoolofMedicine-NorthwellNorthShore/LongIslandJewishMedicalCenter-Manhasset,NY
ZuckerSchoolofMedicine-NorthwellNorthShore/LongIslandJewishMedicalCenter-NewHydePark,NY
ZuckerSchoolofMedicine-NorthwellPlainviewHospital-GreatNeck,NY
ZuckerSchoolofMedicine-NorthwellZuckerHillsideHospital
ZuckerSchoolofMedicine,OM-NorthwellNS/LIJ,Uniondale,NY
ZuckerSchoolofMedicine/NorthwellHealth
ZuckerSchoolofMedicineatNorthwell
ZuckerSOM-Northwell-NY
ZuckerSOM-NorthwellCohenChi
ZuckerSOM-NorthwellCohenChildrens-NY
ZuckerSOM-NorthwellGlenCoveHosp-NY
ZuckerSOM-NorthwellHuntingtonHosp-NY
ZuckerSOM-NorthwellLenoxHillHosp-NY
ZuckerSOM-NorthwellMatherHosp-NY
ZuckerSOM-NorthwellNS/LIJ-N.Y.
ZuckerSOM-NorthwellNS/LIJ-NY
ZuckerSOM-NorthwellNS/LIJ,GreatNeck,NY
ZuckerSOM-NorthwellNS/LIJ,NewHydePark,NY
ZuckerSOM-NorthwellPeconicBay-NY
ZuckerSOM-NorthwellPhelpsHosp-NY
ZuckerSOM-NorthwellPlainviewHosp-NY
ZuckerSOM-NorthwellSouthShore-NY
ZuckerSOM-NorthwellStatenIslandU,N.Y.
ZuckerSOM-NorthwellStatenIslandUniv-NY
ZuckerSOM-NorthwellZuckerHillside-NY
ZuckerSOM-NorthwellZuckerHillside,N.Y.
ZuckerSOM-NorthwellLenoxHillHosp-NY
ZuckerSOM-NorthwellNS/LIJ-NY
ZuckerSOM–NorthwellZuckerHillside–GlenOaks,NY
ZuckerSOMNorthwellNS/LIJNY
ZuckerSOMNorthwellSouthShoreNY

Supplemental Figure 1:

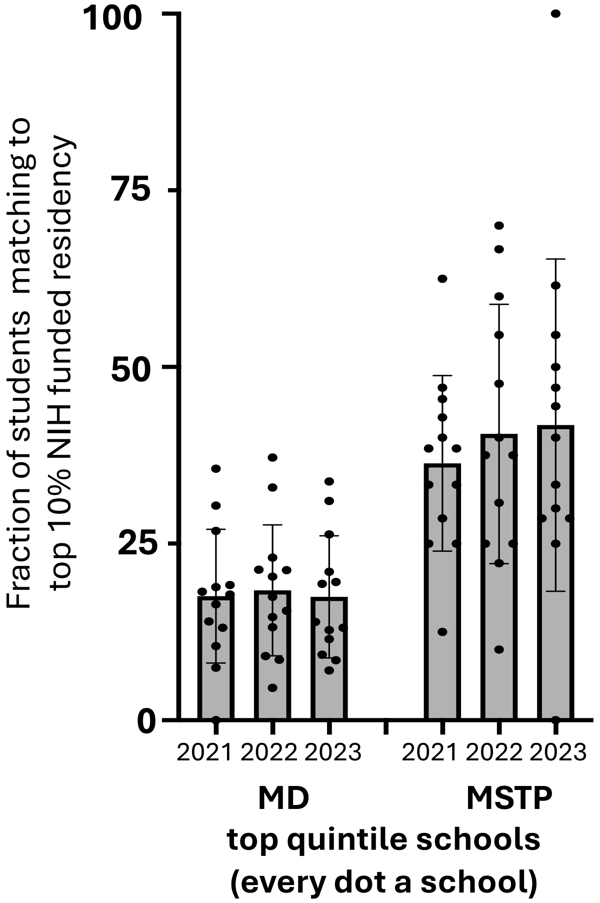

Figure S1. Percent of students in top quintile medical schools matching to top 10%ile residencies, by training program and year. Mean and standard deviation shown. Each dot is a school's average of student matches.

Supplemental Figure 2:

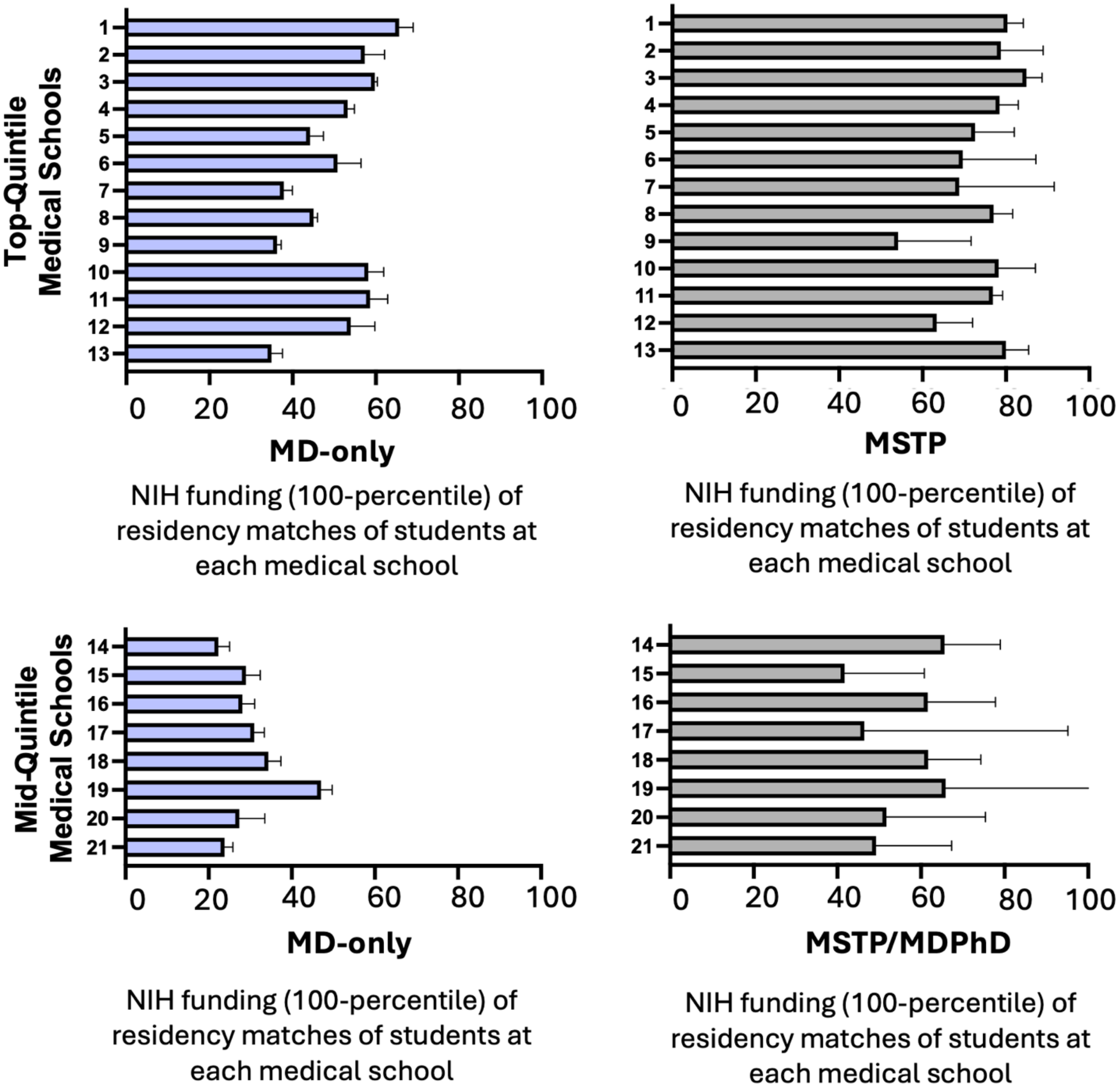

Figure S2. Average match (over 2021-3) of students by training group at top-quintile and mid-quintile schools. Percentile of matched residency calculated by NIH support of matched clinical specialty at institution/year as in text. Mean and Standard deviation shown.

Supplemental Methods

Generation of graphical abstract

We used Google Gemini AI under the Google Workspace for Education Fundamentals license tier to draw the graphical abstract for this article. Accordingly, input into the AI remains private and is not used for model training. The following prompt was used for Google Gemini AI: Please draw a picture. Have a horizontal bar divided into 3 sections: low, mid, and high. The bar should be labeled on the side: NIH funding of matched residency department. Above the bar should be a label: Top-level NIH funded medical schools. Between that label and the bar should be 2 icons: MSTP graduates and MD-only graduates. Icons should be gender and race neutral. Single arrow should go between the MSTP icon and the "high" portion of the bar below. No other arrow should go from that icon above the bar. Arrow should go between MD-only only to the "mid" portion of the bar below. No other arrow should go from that icon above the bar. Arrange so that arrows do not cross. In diagram, MD-only graduates should be on the left and MSTP graduates should be on the right. Below the bar labeled NIH funding of matched residency department there should be another label Mid-level NIH funded medical schools. Right above that label there should be 3 icons labeled MD-only graduates, MDPhD (non NIH-programs), and MSTP graduates (NIH programs). Icons should be gender and race neutral. Arrow should go between this MSTP icon and the "mid" portion of the residency bar above. Single arrow should go between the MD-only icon and the "low" portion of the residency bar above. Single arrow should go between this MDPhD icon and the "mid" portion of the residency bar above. Single arrow should go between the MSTP icon and the “mid” portion. Arrows should not cross: MD-only graduates should be on the left, MDPhD graduates should be in the middle and MSTP graduates should be on the right. The “Top level NIH funded medical school” and “Mid-level NIH-funded medical schools” bars should be the same color such that the text stands out. The middle (NIH funding of matched residency department) bar should be a different color with text standing out. The overall title should be in in bold “**Residency matches by training program, 10668 medical students”** and centered over the bars below. There should be space between the title and the bars. Make extra space between the text box at bottom and the bars. The “Top-level NIH funded medical schools” and “Mid-level NIH funded medical schools” bars should have the same font color and background. There should be no overall fill.

At the very bottom, have a text box with a square around it that reads: Both in total and within schools, MSTP and MDPhD applicants matched to higher NIH-funded residency departments than MD-only applicants, 2021-2023. This text box should be justified on the left with the title. All 3 bars should be centered and boxed. After this is drawn, put a label outside of the diagram to the left of the middle bar that reads “NIH funding of matched residency department. The middle bar should remain centered between the other 2 bars and this label should be separate on the left side of the middle bar. Justify on the left the text box in the middle and the "NIH funding of matched residency department" to match the left edge of the title.
